# Linker histone epitopes are hidden by in situ higher-order chromatin structure

**DOI:** 10.1186/s13072-020-00345-9

**Published:** 2020-06-06

**Authors:** Vladimir B. Teif, Travis J. Gould, Christopher T. Clarkson, Logan Boyd, Enoch B. Antwi, Naveed Ishaque, Ada L. Olins, Donald E. Olins

**Affiliations:** 1 School of Life Sciences, University of Essex, Wivenhoe Park, Colchester, CO4 3SQ UK; 2 Department of Physics & Astronomy, Bates College, Lewiston, ME USA; 3 Present Address: StarBird Technologies, LLC, Brunswick, ME USA; 4 Division of Theoretical Bioinformatics, German Cancer Research Center (DKFZ), Im Neuenheimer Feld 280, Heidelberg, 69120 Germany; 5 Molecular and Cellular Engineering, Centre for Biological Signalling Studies, University of Freiburg, Schänzlestraße 18, Freiburg im Breisgau, 79104 Germany; 6 Charité - Universitätsmedizin Berlin, corporate member of Freie Universität Berlin, Humboldt-Universität zu Berlin, and Berlin Institute of Health, Berlin, Germany; 7 Department of Pharmaceutical Sciences, College of Pharmacy, University of New England, 716 Stevens Avenue, Portland, ME 04103 USA; 8 Digital Health Centre, Berlin Institute of Health (BIH), Anna-Louisa-Karsch-Str. 2, Berlin, 10178 Germany

## Abstract

**Background:**

Histone H1 is the most mobile histone in the cell nucleus. Defining the positions of H1 on chromatin in situ, therefore, represents a challenge. Immunoprecipitation of formaldehyde-fixed and sonicated chromatin, followed by DNA sequencing (xChIP-seq), is traditionally the method for mapping histones onto DNA elements. But since sonication fragmentation precedes ChIP, there is a consequent loss of information about chromatin higher-order structure. Here, we present a new method, xxChIP-seq, employing antibody binding to fixed intact in situ chromatin, followed by extensive washing, a second fixation, sonication and immunoprecipitation. The second fixation is intended to prevent the loss of specifically bound antibody during washing and subsequent sonication and to prevent antibody shifting to epitopes revealed by the sonication process. In many respects, xxChIP-seq is comparable to immunostaining microscopy, which also involves interaction of the primary antibody with fixed and permeabilized intact cells. The only epitopes displayed after immunostaining are the “exposed” epitopes, not “hidden” by the fixation of chromatin higher-order structure. Comparison of immunoprecipitated fragments between xChIP-seq versus xxChIP-seq should indicate which epitopes become inaccessible with fixation and identify their associated DNA elements.

**Results:**

We determined the genomic distribution of histone variants H1.2 and H1.5 in human myeloid leukemia cells HL-60/S4 and compared their epitope exposure by both xChIP-seq and xxChIP-seq, as well as high-resolution microscopy, illustrating the influences of preserved chromatin higher-order structure in situ. We found that xChIP and xxChIP H1 signals are in general negatively correlated, with differences being more pronounced near active regulatory regions. Among the intriguing observations, we find that transcription-related regions and histone PTMs (i.e., enhancers, promoters, CpG islands, H3K4me1, H3K4me3, H3K9ac, H3K27ac and H3K36me3) exhibit significant deficiencies (depletions) in H1.2 and H1.5 xxChIP-seq reads, compared to xChIP-seq. These observations suggest the existence of in situ transcription-related chromatin higher-order structures stabilized by formaldehyde.

**Conclusion:**

Comparison of H1 xxChIP-seq to H1 xChIP-seq allows the development of hypotheses on the chromosomal localization of (stabilized) higher-order structure, indicated by the generation of “hidden” H1 epitopes following formaldehyde crosslinking. Changes in H1 epitope exposure surrounding averaged chromosomal binding sites or epigenetic modifications can also indicate whether these sites have chromatin higher-order structure. For example, comparison between averaged active or inactive promoter regions suggests that both regions can acquire stabilized higher-order structure with hidden H1 epitopes. However, the H1 xChIP-seq comparison cannot define their differences. Application of the xxChIP-seq versus H1 xChIP-seq method is particularly relevant to chromatin-associated proteins, such as linker histones, that play dynamic roles in establishing chromatin higher-order structure.

## Introduction

Histone H1 plays a distinctly different structural and functional role in eukaryotic nuclei compared to the inner (core) histones H4, H3, H2A and H2B. Whereas the inner histones form a defined octamer complex surrounded by a DNA wrapping (the nucleosome), H1 is positioned outside the nucleosome. The central H1 globular domain is at/near the dyad axis, flanked by N- and C-terminal peptide tails, believed to be associated with linker DNA connecting adjacent nucleosomes [1–8]. The inner histones maintain the stability and conformational flexibility of the nucleosome, which represents the fundamental “structural quantum” of chromatin [9, 10]. Histone H1 is essential for maintaining the stability and plasticity of polynucleosomal higher-order structure in vivo. Recent studies suggest that linker histones are acting as a dynamic liquid-like glue for chromatin rather than forming fixed, stable complexes with nucleosomes [11, 12].

The stoichiometry of histone H1 per histone octamer has been estimated to be ~ 0.8–1.0 in somatic cells [13, 14]. Generally, six isotypes (variants) are observed in somatic human cells: H1.0, H1.1, H1.2, H1.3, H1.4 and H1.5 [5, 15–17]. In vitro, the presence of histone H1 is required to condense polynucleosomal chains at physiological ionic strength. The C-terminal tail of histone H1 is more important to the formation of chromatin higher-order structure than is the N-terminal tail, which still plays a role [4, 18]. Genetic loss of certain histone isotypes can apparently be compensated by H1 isotype redundancy, until the stoichiometry of H1/nucleosome becomes too low, resulting in embryonic lethality, possibly due to chromatin decompaction [14].

Several studies have examined the in situ enrichment (or depletion) of DNA functional elements at the binding sites of various H1 isotypes [19–22]. These studies employed H1 xChIP-seq on a variety of undifferentiated and differentiated cells, fixed with formaldehyde, followed by sonication and subsequent immunoprecipitation. It has been argued that in differentiated cells, H1.5 is associated with compacted heterochromatin, involved with repression of transcription and does not overlap enhancers [22]. In addition, data have been published that H1.2 and H1.3 are depleted from GC- and gene-rich regions, active promoters and transcription start sites (TSS); but enriched in AT-rich regions and “gene deserts” [19]. H1.2 has been described as “showing the most specific pattern and strongest correlation with low gene expression” [21]. It has also been stated that H1.2 and H1.5 are depleted from CpG-dense regions and active regulatory regions [20]. The authors of the latter study argue that there is an overrepresentation of depleted regions of all H1 subtypes at promoters.

**Fig. 1:**
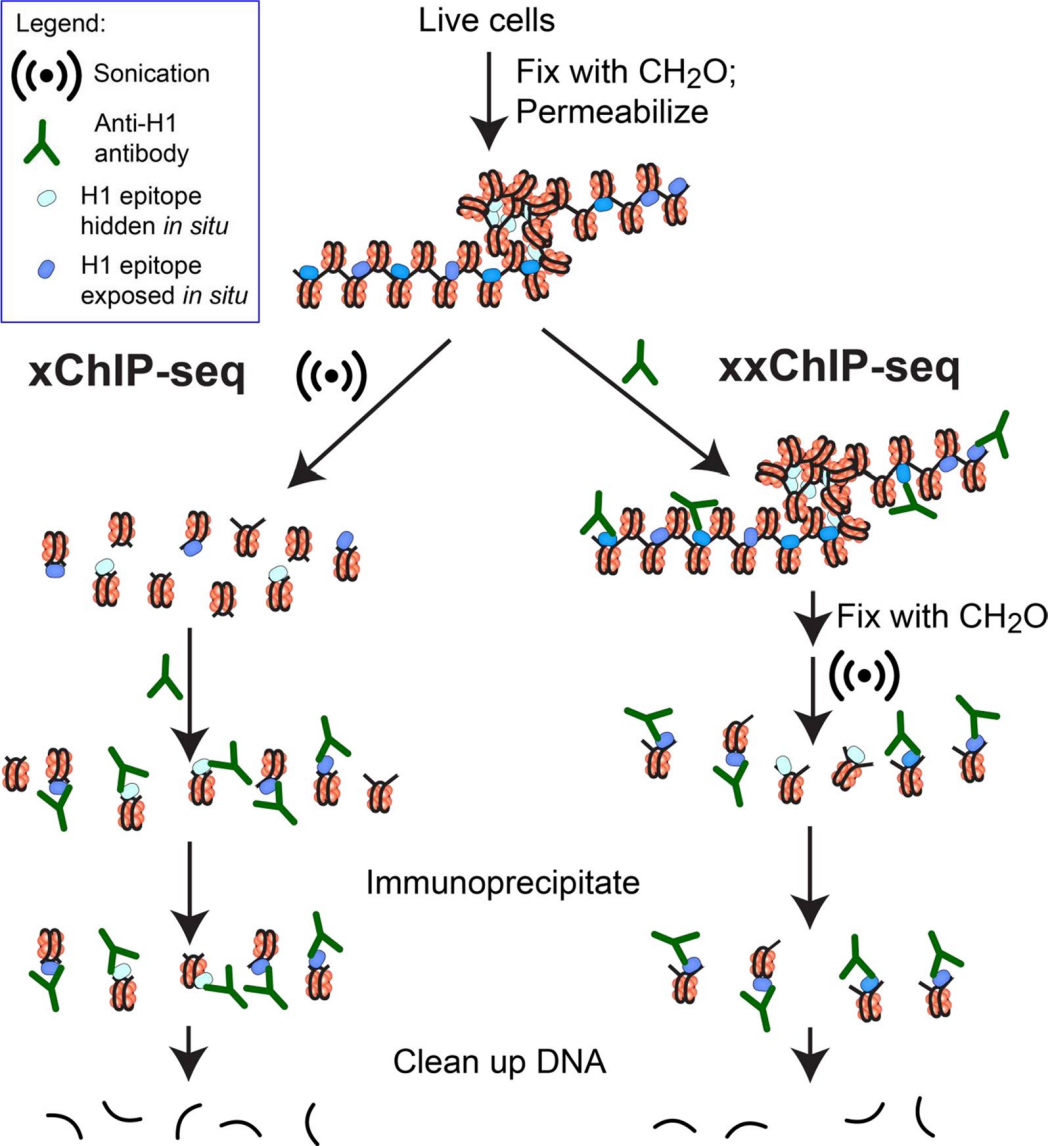
A scheme explaining the difference between the xChIP-seq and xxChIP-seq protocols. Note that the DNA sequences that are associated with “hidden” epitopes in situ are revealed by xChIP-seq, but not with xxChIP-seq. This scheme does not specify the type of chromatin higher-order structure involved in creating the “hidden” H1 epitope. “Masking” proteins, capable of “blocking” the H1 epitope in vivo, certainly can exist. Probably, the first formaldehyde fixation will covalently attach these proteins to H1 (and mask the epitope), such that the sonicated products cannot be immunoprecipitated at any point in the protocol. The xxChIP-seq versus the xChIP-seq comparison depends upon the differences in “exposed” H1 epitopes. If the masking protein is “knocked-off” during the xChIP sonication, exposing the H1 epitope, it will “mimic” higher-order structure, which is (presumably) also destroyed during the xChIP sonication, exposing H1 epitopes. “Higher-order chromatin structure” is only detected after anti-H1 binding, washing, a second formaldehyde fixation and sonication (xxChIP). Any exposure of H1 epitopes, at this point, will be undetected, since there is no further incubation with anti-H1 antibodies. Immunoprecipitation occurs because the Protein A/G agarose captures chromatin fragments by their covalently bound IgG (anti-H1) molecules

In the present study, the chromatin distributions of two isotypes (H1.2 and H1.5) were examined within the nuclei of the human myeloid leukemia cell line HL-60/S4 in situ. Two chromatin immunoprecipitation methods were employed, and their results compared; i.e., xChIP-seq and xxChIP-seq, see Fig. 1 for a schematic explanation of these two methods. In the standard xChIP-seq method, formaldehyde-fixed and permeabilized cells are sonicated to nucleosome-size fragments, prior to incubation with antibody and immunoprecipitation. In the newer xxChIP-seq method [23], which was designed to “parallel” immunostaining microscopy, fixed and permeabilized cells are incubated with primary antibody, washed and fixed a second time, prior to sonication and immunoprecipitation. The second fixation is intended to prevent the loss or shifting of specifically bound antibody during the washing and processing of antibody-bound fragments. Thus, while information about the influence of chromatin higher-order structure on H1 distribution and epitope exposure is lost using xChIP-seq, it is preserved in xxChIP-seq. A comparison of similarities and differences between the results of xChIP-seq and xxChIP-seq, employing anti-H1.2 and H1.5, provokes speculations about the possible influences of in situ chromatin higher-order structure and function upon H1 epitope exposure.

## Materials and methods

### Cell culture and antibodies

The human myeloid leukemia cell line HL-60/S4 (ATCC, CRL-3306) was maintained in RPMI-1640 medium plus 10% FCS and 1% Pen/Strep/Glut. Cells were grown in 6 ml of medium in T-25 flasks, generally split (1:6–1:12) 2–3 times/week. For large-scale preps, cells were grown in T-75 flasks with up to 30 ml media. Cell concentrations were monitored using a hemocytometer. Rabbit polyclonal ChIP-grade antibodies were obtained from Abcam: anti-histone H1.2 (ab4086) and anti-H1.5 (ab18208). Both antibodies are directed against antigenic determinants within the N-terminal 1-100 aa residues.

### Immunostaining and STED imaging

Images of immunostained cells were obtained on a Leica SP8 confocal microscope and on a home-built STED microscope, as described previously [24]. H1.2 and H1.5 domain sizes were determined using custom-written software in Matlab to analyze STED/confocal image pairs acquired on our STED microscope. Domains in confocal images were user-identified and fit to a two-dimensional (2D) Gaussian intensity profile to determine the full-width at half-maxima (FWHM) in the X- and Y-directions. For STED images, domains were programmatically segmented starting with the brightest (i.e., the highest summed intensity in a local region of interest) domains and fit to a 2D Lorentzian intensity profile to determine FWHM. Lorentzian fits were rejected if the fitting routine resulted in a negative amplitude, a center location outside the local region of interest, or if the fractional uncertainty in the fitted width was greater than 60%. In a given STED image, the domain segmentation was terminated whenever 50% of the 20 most recent attempted Lorentzian fits were rejected. The distribution of domain sizes was generated using the average X and Y FWHM for each fitted domain.

### ChIP-seq

All ChIP-Seq experiments were performed on undifferentiated HL-60/S4 cells that were fixed, permeabilized and stored in cryovials (containing ~ 10^7^ cells/cryovial) in liquid Nitrogen. Prior to storage, the cells were harvested from growth medium at ~ 10^6^ cells/ml, centrifuged and washed with PBS, fixed in 1% HCHO/PBS for 10 min at room temperature (RT), stopped with 0.125 M glycine for 5 min, washed with PBS, followed by PBS + 0.1 M PMSF. The fixed cells were permeabilized for 10 min at 4 °C in a lysis buffer containing 25 mM HEPES buffer (pH 7.8), 1 mM MgCl_2_, 10 mM KCl, 0.1% NP40, 1 mM DTT and 0.5 mM PMSF. Following centrifugation and removal of supernatants, cell pellets were frozen in residual lysis buffer at liquid Nitrogen temperature.

For both single-fixation and double-fixation ChIP (xChIP and xxChIP, see Fig. 1), chromatin was disrupted with a Covaris Focused Ultrasonicator M220. In xChIP, each frozen cell pellet (1 cryovial) was dispersed in 130 µl of Covaris Sonication Buffer (1 mM EDTA, 10 mM Tris [pH 7.6], 0.1% SDS), followed by sonication (20 min, 200 cycles, 75 Watts, Duty Cycle 20%, 7 °C). The sonicates were centrifuged at 18,000xg, 10 min, 4 °C and the supernatants recovered. SDS was reduced in the supernatants to ~ 0.003% and replaced with 0.05% Tween-20, employing repeated dilution with PBST (PBS + 0.05% Tween-20) and centrifugal concentration using a Centricon YM-50. Six centrifugations of ~ 1/2 dilutions with PBST at 1000×*g*, 10 min resulted in ~ 0.5 ml of the final retentate with reduced SDS. IgG-free BSA (Sigma A3294) was added to a final BSA concentration of 5%.

In xxChIP, the once-fixed frozen cell pellets were dispersed in a buffer reminiscent of the permeabilizing buffer used in immunostaining reactions (0.1% Triton X-100, 0.1 mM PMSF plus Sigma Protease Inhibitor Cocktail [P8340]) for 20 min at RT. After PBS washes, the permeabilized cells were suspended in PBST + 5% IgG-free BSA (PBSTB) and rotated for 90 min at RT. To 300-µl aliquots containing ~ 6 × 10^7^ cells, the primary antibody was added: 30 µl anti-histone H1.2 (1 mg/ml) or 60 µl anti-histone H1.5 (0.5 mg/ml). The cells plus antibody were rotated for 4 h at RT. Following antibody incubation, the cells were washed several times with PBS to remove unbound antibody. For the second fixation, the washed cells were made 1% HCHO/PBS and rotated 2.5 min at RT. Fixation was stopped with 0.125 M glycine for 5 min, cells washed with PBS and dispersed in 1.0 ml of Covaris Sonication Buffer (1 mM EDTA, 10 mM Tris [pH 7.6], 0.1% SDS), followed by sonication at optimized conditions (40 min, 400 cycles, 75 Watts, Duty Cycle 26%, 7 °C). The sonication buffer was replaced with PBST, employing centrifugal concentration, as described above.

Of necessity, the immunoprecipitation (IP) protocols differed slightly, comparing xChIP to xxChIP. The xChIP preparations in PBSTB buffer were incubated with control agarose (1 h, with rotation) and recovered from the minicolumns by centrifugation at 4 °C. Samples of these “cleaned” sonicates were retained as “Input”. Simultaneously, Protein A/G agarose minicolumns, equilibrated in PBSTB, were incubated for 4–5 h with 4 µg of rabbit anti-histone H1.2 or H1.5, rotating at RT, followed by washing with PBSTB. The equilibrated and “cleaned” sonicates were incubated with the antibody-bound Protein A/G agarose minicolumns overnight, rotating at 4 °C. Subsequently, the sonicate-bound columns were washed 5 times with PBSTB and 2 times with PBST, to remove unbound chromatin. Elution of the bound chromatin fragments was accomplished by addition of 50 µl of 100 mM NaHCO_3_ + 1% SDS, tumbling for 15 min at RT. After centrifugal recovery, a second elution with 50 µl was performed, yielding ~ 100 µl of pooled eluate. The IP eluates were digested with RNAse and proteinase K, overnight at 65 °C. DNA was purified employing Sigma Gene Elute (NA1020-1KT). By contrast, xxChIP preparations, having the anti-H1 antibodies already bound and crosslinked to the chromatin fragments and in PBSTB buffer, were “cleaned” on the control agarose minicolumns, an aliquot removed for “Input” and the remainder incubated with Protein A/G agarose minicolumns overnight, rotating at 4 °C, followed by washing to remove unbound chromatin. Elution of the bound chromatin fragments and DNA purification were similar to the xChIP procedures.

### ChIP-seq analysis

xChIP-seq and xxChIP-seq with antibodies against histone H1.2 and H1.5 were each performed in triplicate. Paired-end sequencing was conducted by the sequencing facility of the German Cancer Research Center (DKFZ) using Illumina HiSeq 2000 and processed with manufacturer’s software HCS 2.2.58 and RTA 1.18.64. xChIP and xxChIP sequencing data were aligned to the human genome hg19 using Bowtie2 [25] allowing up to 2 mismatches and accepting only uniquely mappable reads. Regions enriched with H1.2 and H1.5 binding were determined by peak calling with MUSIC [26] using default parameters. We determined ~ 60,000–70,000 peaks per replicate per condition and then merged the peaks for all triplicates within each condition. Regions dominated by H1.2 over H1.5 (and vice versa) were determined using NucTools [27] for a 100-bp sliding window, considering only windows with relative standard deviation between the three replicates within each condition < 0.5 and the relative difference between the average occupancy of H1.2 and H1.5 > 0.99. Chromosome-wide signals were visualized using the IGV genome browser with 1000-bp smoothing window. Fold enrichment of signals in genomic regions were calculated using BedTools commands *intersectBed* and *shuffle* [28] as a ratio of the observed number of regions overlapping between two features of interest to the number of overlapping regions expected by chance. Average aggregate profiles were calculated using HOMER [29]. The profile plotted is the value of the xChIP or xxChIP signal divided by the corresponding Input.

### External datasets

The whole-genome bisulfite sequencing reported in our recent study [30] is available in the ENA database under accession PRJEB27665. ChIP-seq of histone H3, epichromatin, and histone modifications reported in our previous publication [31] are available in the GEO database (GSE90992). ChIP-seq datasets of Pol 2, H3K27ac, H3K4me1 and ERG1 in HL-60 cells published in [32] were kindly provided by Marco Trizzino in the form of BED files with peaks (hg19 genome assembly). ChIP-seq datasets of REST, CTCF, GABPA, JMJD1C, SMC3, SPI1 and STAG1 in HL-60 cells were obtained from the ReMap database [33] in the form of BED files with peaks (hg19). Coordinates of DNase I-sensitive regions determined by the ENCODE consortium in HL-60 cells [34] were obtained in the form of BED files (hg19) from the GEO database (GSM736595), and two replicates were merged together.

## Results

### Immunostaining of interphase nuclei with anti-H1.2 and H1.5 demonstrates punctate structures

**Fig. 2:**
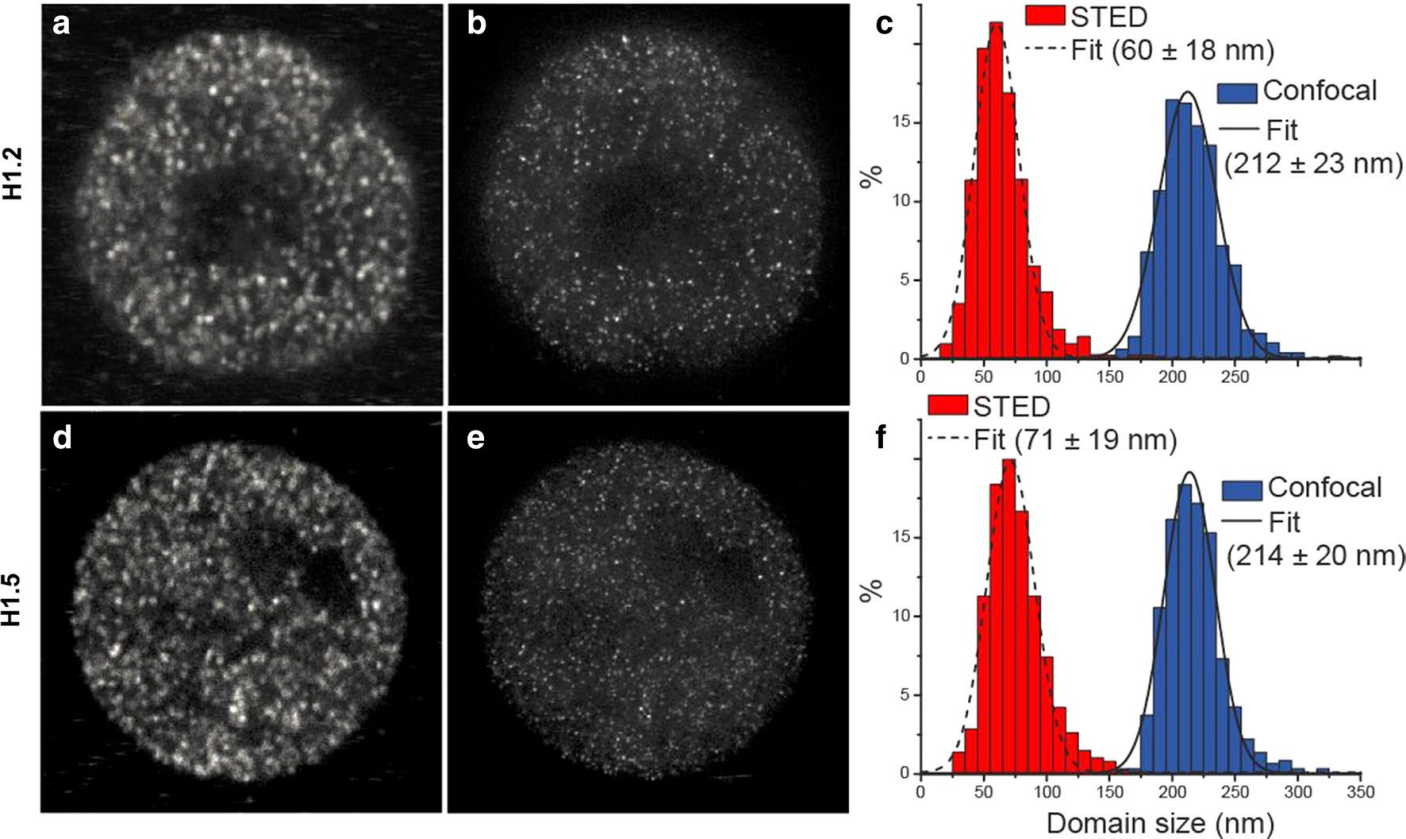
Images and statistics of size distributions of H1.2 (**a**–**c**) and H1.5 (**d**–**f**) punctate chromatin domains (“chromomeres”) based on confocal (**a** and **d**) and STED (**b** and **e**) microscopy. Cells were fixed with HCHO, permeabilized with Triton X-100/PBS and blocked with 5% normal goat serum/PBS prior to immunostaining, as described earlier (24)

Previous studies [12, 24] have revealed that punctate chromatin structures (“chromomeres”) can be observed within fixed and permeabilized interphase nuclei and mitotic chromosomes of HL-60/S4 cells by immunostaining with bivalent rabbit anti-histone H1.5. Similar punctate structures were also observed in HL-60/S4 cells employing the monovalent Fab fragment from the mouse mAb PL2-6, an autoimmune antibody directed against the nucleosome “acidic patch” (consisting of acidic amino acid residues from histones H2A and H2B) [12, 24, 35, 36]. Figure 2a–f and Additional file 1: Figure S1 present images of undifferentiated HL-60/S4 interphase nuclei immunostained with rabbit anti-H1.2 and with rabbit anti-H1.5. The chromomeric patterns are readily visible by both confocal and STED microscopy. Employing stimulated emission depletion (STED) microscopy yielded an estimate of the diameters of H1-enriched foci (anti-H1.2, ~ 60 nm; anti-H1.5, ~ 70 nm), approximately threefold smaller than the diameters estimated by confocal imaging (~ 210 nm). As stated earlier [12, 24], we suggest that these chromomeres may represent the fixed and stained equivalent of constrained polynucleosome clusters observed by a variety of biochemical and microscopy methods (e.g., Hi-C, replication foci and TIRF microscopy). The punctate immunostaining pattern of H1 epitope distribution likely reflects an in situ chromatin higher-order organization within fixed interphase nuclei.

### Chromatin immunoprecipitation (IP) with anti-H1 demonstrates differences in genomic element enrichment/depletion

Currently, analysis of the genome-wide distribution of DNA-binding proteins (unmodified or modified by post-translational changes) is frequently performed by chromatin immunoprecipitation followed by DNA sequencing (ChIP-seq). Usually the intact cells are fixed once with formaldehyde, followed by sonication to nucleosome-size fragments and subsequent IP (xChIP). However, information about the influence of in situ chromatin higher-order structure on protein (epitope) distribution is largely lost during the sonication step, which precedes the IP step. We developed a modified ChIP-seq protocol to preserve information about the influence of in situ chromatin higher-order structure on protein (epitope) distribution. The method involves two formaldehyde fixation steps: the first, of intact cells; the second, following permeabilization and antibody binding, but before sonication, IP and DNA sequencing (xxChIP-seq) [23]. A scheme comparing xChIP-seq and xxChIP-seq is shown in Fig. 1, for the specific situation of mapping the distribution of histone H1. This figure contrasts H1 epitopes that are “exposed” in situ and H1 epitopes that are “hidden” by bound protein and/or chromatin higher-order structure “masking”. The xxChIP-seq method was originally developed to define the DNA sequences within “epichromatin”, the surface of chromatin beneath the nuclear envelope by employing the bivalent mAb PL2-6 [12, 23, 24, 37]. In this situation, the epichromatin epitope, which is present on all nucleosomes, is largely “hidden” internally and “exposed” at the chromatin surface. Performing both methods (xChIP-seq and xxChIP-seq) on the same cell type can furnish a detailed genome-wide comparison of “exposed” versus “hidden” epitope regions.

Employing xChIP-seq and xxChIP-seq with rabbit anti-histone H1.2 and H1.5 antibodies, we determined genome-wide distributions of these signals and using peak calling software MUSIC [26] determined the regions (peaks) with their enrichments. The average size of such peaks was around 2000 bp (Figure S2). xChIP H1.2 and xxChIP H1.2 and H1.5 were characterized on average by a slight depletion of GC content at about 500 bp from the peak summit, whereas in the case of xChIP H1.5, the peaks did not have any pronounced nucleotide signature (Additional file 1: Figure S3).

**Fig. 3:**
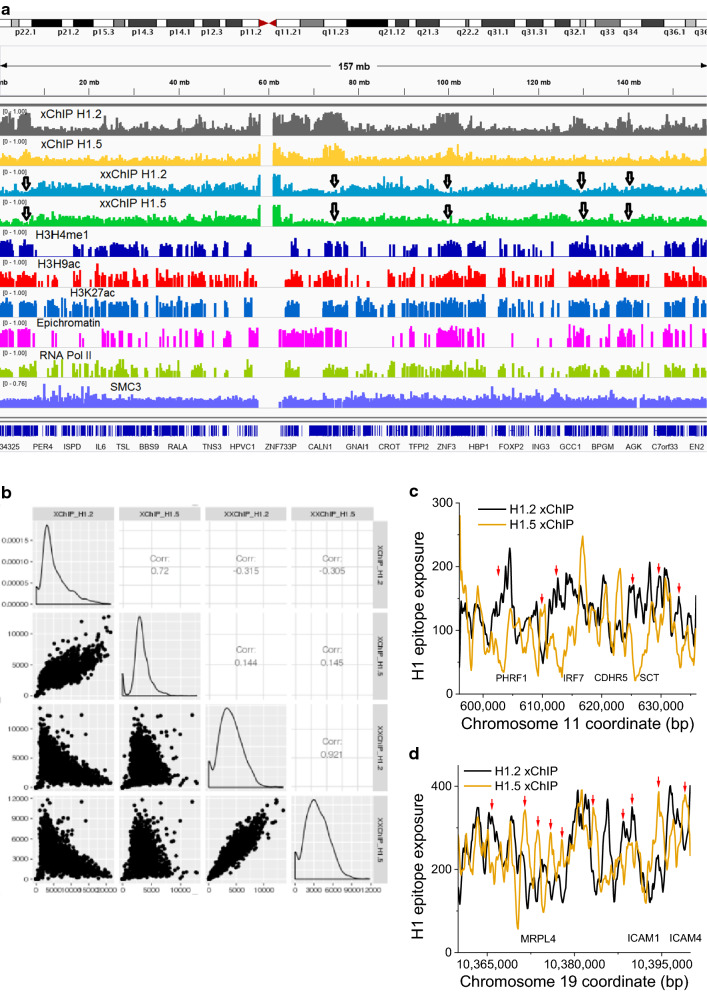
Large-scale (low resolution) and small-scale (high resolution) comparisons between xChIP and xxChIP sequencing strategies. **a** H1 epitope “exposure” peak densities over chromosome 7, as measured by scanning with a window of 1000 base pairs: for xChIP H1.2 (dark blue), xChIP H1.5 (magenta), xxChIP H1.2 (orange) and xxChIP H1.5 (green). Black arrowheads show enrichments (compared to surrounding regions) of xChIP-seq reads for H1.2 and H1.5, coupled with deficiencies for xxChIP-seq reads of H1.2 and H1.5 at the same locations. Also indicated are cytogenetic road marks, DNA lengths (mb), the epichromatin track [23], and tracks for the density of peaks enriched with H3K4me1, H3K9ac, H3K27ac, Pol II and SMC3. **b** Pairwise correlations between xChIP H1.2, xChIP H1.5, xxChIP H1.2 and xxChIP H1.5 signals, averaged over three replicates each, using a 1000-bp sliding window. Note that xChIP H1.2 versus xChIP H1.5 and xxChIP H1.2 versus xxChIP H1.5 reveal clear positive correlations; whereas, the xChIP signals reveal negative correlations with the xxChIP signals. **c** and **d** Examples of genomic regions at high resolution, showing distinct patterns of histone occupancy for aligned reads of H1.2 (black) and H1.5 (orange) xChIP-seq. The raw xChIP-seq signal was smoothed by averaging with a 100-bp running window

Figure 3a presents parallel tracks along human chromosome 7, illustrating the density of peaks enriched for histones H1.2 and H1.5 xChIP-seq and xxChIP-seq, as well as several other epigenetic signals measured in HL-60/S4 cells [23, 31] (see all other chromosomes in Additional file 1: Figure S4). Figure 3a illustrates interesting correlations among several tracks. In the regions denoted by black arrowheads, there are more (compared to surrounding regions) of xChIP-seq of domains enriched with H1.2 and H1.5, coupled with deficiencies for xxChIP-seq domains enriched with H1.2 and H1.5. This type of behavior might signify the presence of “hidden” H1 epitopes within the xxChIP-seq reads of H1.2 and H1.5, possibly due to the presence of higher-order chromatin structure (Fig. 1). Many of these regions also correlate with enrichments of H3K4me1, H3K9ac, H3K27ac and RNA Pol II, all markers of transcription-permissible regions (based on published ChIP-seq, see Methods). Perhaps the formaldehyde-fixed transcriptional apparatus (transcription factories?) generates steric “hiding” of H1 epitopes in the xxChIP-seq assay. A systematic correlation analysis comparing xChIP-seq and xxChIP-seq (with themselves and each other) is presented in Fig. 3b. It is of interest that H1.2 xChIP and H1.5 xChIP reveal a positive correlation at the kb scale, and that H1.2 xxChIP and H1.5 xxChIP demonstrate even better correlation. On the other hand, the xChIP signals reveal negative correlation with the xxChIP signals. This latter observation supports the view that fixation-preserved in situ higher-order chromatin structure results in a significant fraction of “hidden” H1 epitopes.

As it is clear from Fig. 3b, genome-wide H1.2 and H1.5 xChIP signals are positively correlated. However, in a number of important regulatory locations we observed mutually excluding arrangements of H1.2 and H1.5. Such examples, shown in Fig. 3c, d, are characterized by “swings” of 3–5 nucleosomes in H1.2 and H1.5 linker histone enrichment, with some regions exhibiting H1 isotype predominance for longer distances. We then set to define the locations of all such regions using NucTools with a sliding window of 100 bp. Interestingly, many such regions were at gene promoters. In particular, this analysis revealed 1715 regions where xChIP H1.2 dominates over H1.5 (39.8% of them at promoters), and 5214 regions where xChIP H1.5 dominates over H1.2 (44.2% of them at promoters). Thus, thousands of gene promoters are enriched either with H1.2 or H1.5, suggesting that differential binding of H1 variants has functional implications. Gene Ontology analysis revealed that promoters with H1.2 or H1.5 dominance were enriched for genes related to ATP binding and enzymatic activity (Additonal file 2: Table ST1 and ST2).

**Fig. 4:**
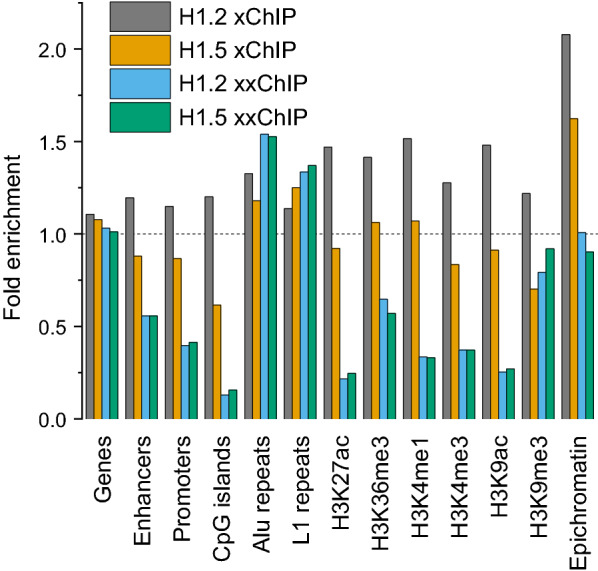
Enrichment/depletion of different genomic features within anti-H1.2 and anti-H1.5 immunoprecipitated domains determined with MUSIC peak calling. Fold changes (Y-axis) above 1.0 indicate enrichment, compared to the genomic average; below 1.0, indicate depletion. Indicated are several histone post-translational modifications (PTMs) and their usual functional associations: H3K27ac and H3K4me1, active enhancers; H3K4me3, active promoters; H3K9ac, active regions; H3K9me3, heterochromatin; H3K36me3, gene body of actively transcribed genes

Next, we analyzed the genome-wide distribution of regions enriched with xChIP and xxChIP H1 signals in relation to different genomic features defined using our previous ChIP-seq of histone modifications in HL-60/S4 cells (25). Figure 4 presents a summary of the relative enrichment (or depletion) of various chromatin features with H1.2 and H1.5 peaks determined by MUSIC peak calling based on xChIP-seq and xxChIP-seq. Some of the conclusions: (1) For most of the studied features, H1.2 xChIP displays more enrichment, than H1.5 xChIP. For example, H1.2 xChIP shows more enrichment of enhancers, promoters, CpG islands, Alu repeats, H3K27ac, H3K36me3, H3K4me1, H3K9ac, H3K9me3 and epichromatin. (2) xxChIP H1.2 and H1.5 signals resemble each other more than xChIP H1.2 and H1.5. (3) xxChIP generally shows more depletion of the studied chromatin features, than observed with xChIP, except for Alu and L1 repeats. To some extent, the differences observed comparing H1.2 and H1.5 xChIP are obliterated when comparing H1.2 and H1.5 xxChIP. (4) Reminiscent of conclusions derived from the chromosome tracks displayed in Fig. 3a, transcription-related regions and “active” histone modifications (i.e., enhancers, promoters, CpG islands, H3K4me1, H3K4me3, H3K9ac, H3K27ac and H3K36me3) are enriched in H1.2 and H1.5 xChIP-seq and show significant depletions in H1.2 and H1.5 xxChIP-seq reads. These observations support that in situ chromatin higher-order structures, “preserved” by formaldehyde fixation, can create “hidden” histone H1.2 and H1.5 epitopes. They also suggest that transcription-related regions may have their own higher-order structure.

### Differential enrichments of H1 variants around protein-binding sites

**Fig. 5:**
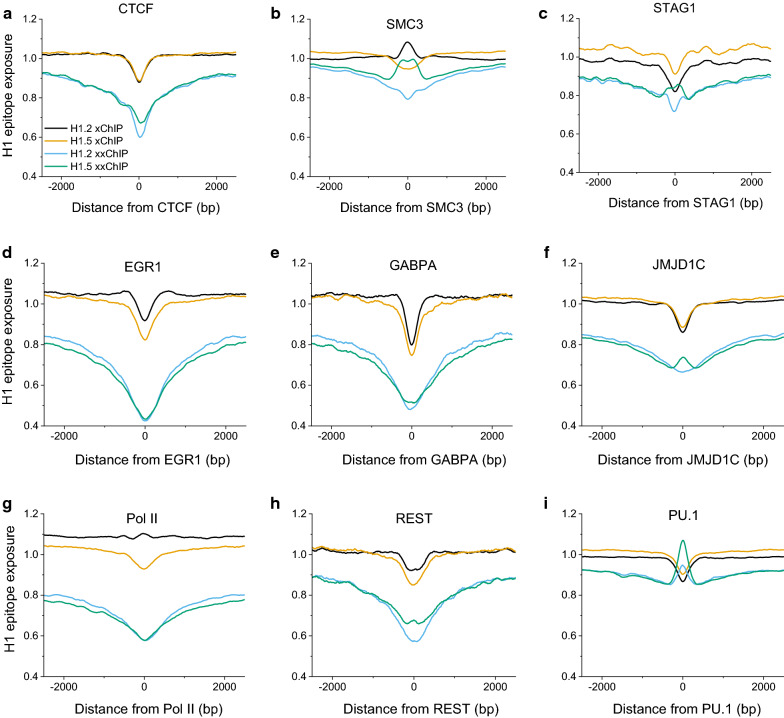
Profiles of H1 epitope exposure in HL-60/S4 cells, centered around different protein-binding sites on DNA as defined by ChIP-seq in HL-60 cells (see Methods). The different DNA-binding proteins/functions: CTCF defines chromosome loops; SMC3, subunit of cohesin; STAG1, subunit of cohesin; EGR1, transcription factor (TF); GABPA, TF; JMJD1C, histone demethylase; Pol II, RNA polymerase; REST, TF; SPI1, TF. Note that the xxChIP profiles for H1.2 and H1.5 “track” together, which always display reduced H1 epitope exposure around the center of the binding site (0). Generally, the xChIP profiles track together, sometimes in the same direction as the xxChIP profiles (EGR1, GABPA, JMJD1C, REST and SPI1); sometimes in the opposite direction (CTCF and STAG1). Interestingly, both Pol II and SMC3 indicate a divergence of the xChIP profiles around the center of the binding site

The apparent occlusion of H1 epitopes, due to the preservation of chromatin higher-order structure surrounding various chromatin protein-binding sites, is presented in Fig. 5. In the case of CTCF-binding regions, H1 xChIP-seq profiles show weak oscillations that have been previously reported in a number of nucleosome positioning studies [38–40]. Interestingly, H1.2 and H1.5 variants are not distinguishable in this case. All other protein-binding regions, presented in this figure, have distinct xChIP-seq profiles for H1.2 and H1.5. In addition, H1 xxChIP-seq profiles around CTCF-binding sites show strong depletion compared to the H1 xChIP-seq profiles, suggesting that, in these localized fixed in situ chromatin regions, H1 epitopes are “hidden” due to stabilization of higher-order structure. A similar clear depletion of H1 xxChIP-seq signals, compared to H1 xChIP-seq signals, was observed for other chromatin-binding proteins (e.g., EGR1, GABPA, JMJD1C, Pol II and REST). In terms of the differences of the profile shapes between H1.2 and H1.5, two chromatin-binding proteins stand out: the subunits of cohesin SMC3 and STAG1. Their xxChIP-seq profiles show differences between H1.5 and H1.2 close to the center of the SMC3- and STAG1-binding sites, suggesting differential roles of these H1 variants in interactions with cohesin. For these selected chromatin protein-binding regions, formaldehyde fixation appears to make H1 epitopes (whose presence is demonstrated in the xChIP-seq) inaccessible to antibody in the xxChIP-seq assay. Another case of a very pronounced difference is observed around PU.1-binding sites. Our previous analysis showed that in HL-60/S4 cells PU.1 is associated with highly ordered nucleosome arrays with ~ 10 bp smaller nucleosome repeat length than genome-average [31]. A recent publication noted that PU.1 acts as a non-classical pioneer factor (not able to bind DNA in the nucleosome, but recruiting remodellers that redistribute nucleosomes) [41]. It seems that this nucleosomal organization exposes H1 epitopes in such a way that the xxChIP signal goes up.

### “Open” chromatin regions have narrow xChIP and wide xxChIP depletion

**Fig. 6:**
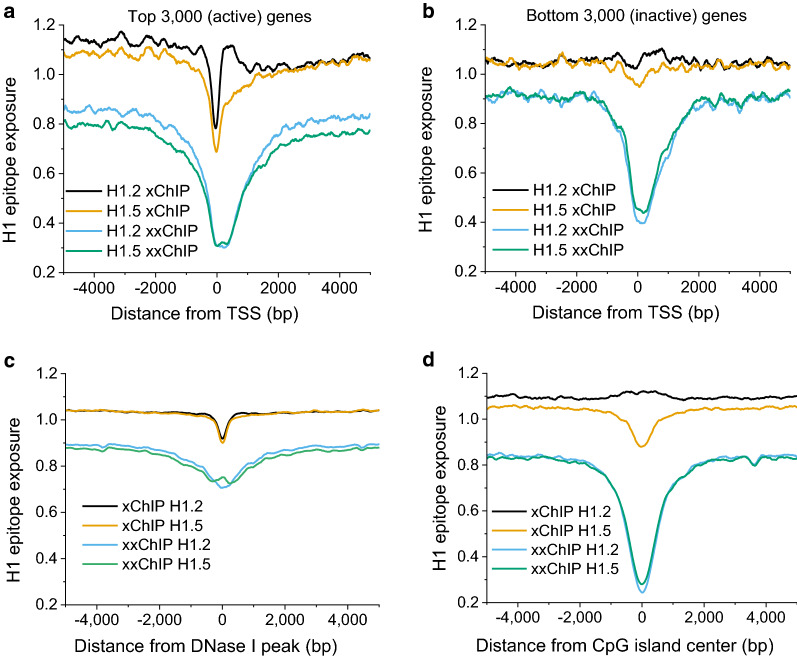
Average H1 epitope exposure in undifferentiated HL-60/S4 cells around transcription start sites, “open” chromatin regions and CpG islands. **a**, **b** xChIP and xxChIP signals around TSS for two groups of genes, sorted by their normalized expression in HL-60/S4 cells (Teif et al.[ 31]): 3000 genes with expression at the top percentile (**a**) and 3000 genes with expression at the bottom percentile (**b**). **c** xChIP and xxChIP H1 profiles around “open” chromatin regions (ENCODE DNase I hypersensitive regions in HL-60 cells) [34]. **d** xChIP and xxChIP H1 profiles around CpG islands. Both xChIP and xxChIP datasets reveal an average “dip” in H1 epitope exposure around the middle of TSS regions; but, clearest with the top percentile genes. The “dip” observed with xChIP may represent the occupancy paucity of H1 in the TSS; xxChIP may emphasize an additional steric “hiding” of the H1 epitopes by proteins involved in active gene expression

Figure 6 presents average H1 epitope exposure around transcription start sites (TSS) for active and inactive genes. Active genes (Fig. 6a) show a significant difference between xChIP and xxChIP. The xChIP profiles contain a sharp and deep decline in apparent H1 occupancy (~ 300 bp wide), corresponding to the nucleosome-depleted region adjacent to the TSS. On the other hand, the xxChIP H1 epitope depletion extends to a ~ 10-fold longer region near the TSS, encroaching onto the gene body, suggesting an extended stabilized higher-order structure. In terms of the differences between H1.2 and H1.5, the epitope depletion of H1.5 is stronger, compared to H1.2. For inactive genes (Fig. 6b), H1 xChIP-seq profiles are essentially unchanged across the TSS regions. The “broad depletions” seen with both H1.2 and H1.5 xxChIP-seq suggest that higher-order chromatin structure is a common feature of TSS regions, regardless of transcriptional activity. It could be that the depletion of xxChIP signal at inactive promoters reflects the decrease of their in situ accessibility.

We then analyzed xChIP profiles around “open” chromatin regions in general. Figure 6c shows average xChIP and xxChIP profiles around DNase I-sensitive sites in HL-60 cells, which are consistently with Fig. 6a. The profiles around CpG islands (Fig. 6d) show the largest depletion of xxChIP, consistently with Fig. 4. Interestingly, xChIP H1.2 and H1.5 profiles around CpG islands are significantly different from each other, consistently with our finding that most regions with “swings” of H1.2 or H1.5 xChIP investigated in Fig. 3c and d are located inside promoters. Collectively, this analysis supports the concept that chromatin regions which are traditionally believed to be “open” generally possess chromatin higher-order structure, which when fixed with formaldehyde in vivo, results in decreased histone H1 epitope exposure.

**Fig. 7:**
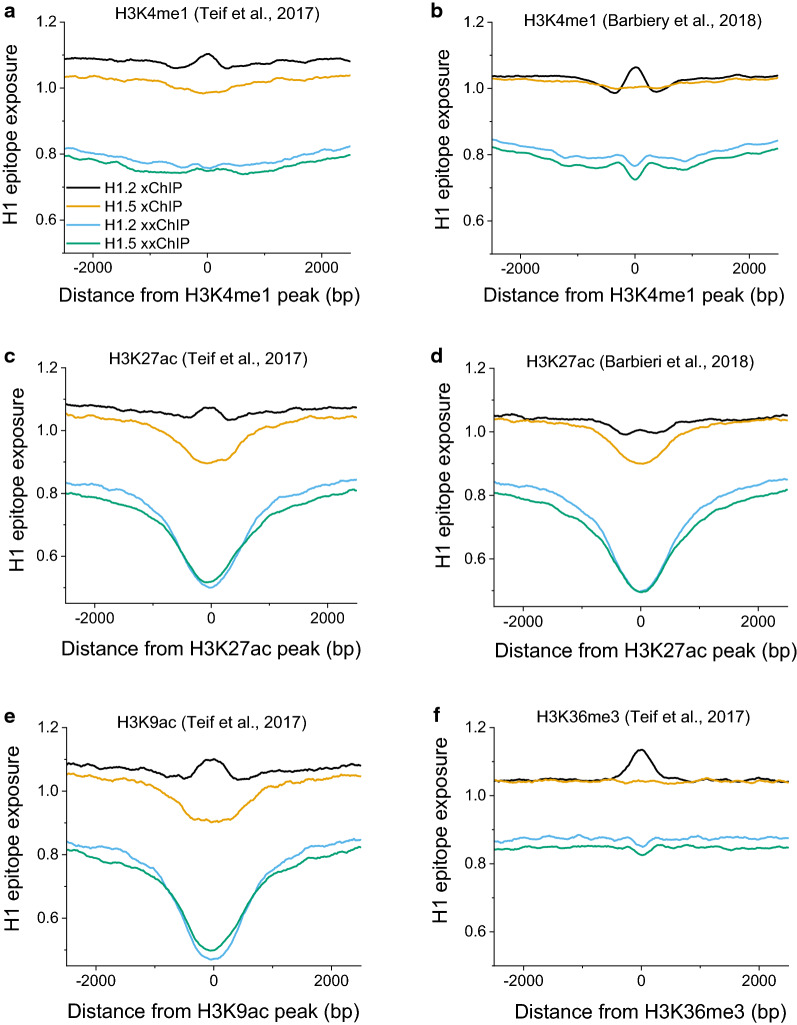
H1 epitope exposure in undifferentiated HL-60/S4 cells surrounding average genomic regions enriched for different histone modifications. Histone modifications have been mapped using ChIP-seq data in HL-60/S4 cells (Teif et al. [31] or HL-60 cells (Barbieri et al. [32]), as specified in the figure. Both H3K4me1 and H3K27ac are usually associated with active enhancers

Figure 7 presents average H1 epitope exposure profiles surrounding genomic regions enriched with different histone modifications. For verification, we have plotted profiles for H3K4me1, H3K27ac, H3K9ac and H3K36me3, using both the data that we reported for HL-60/S4 cells (25), as well as recent data for H3K4me1 and H3K27ac in HL-60 cells [32]. For H3K4me1 (mark of active enhancers), K3K27ac and H3K9ac (general activating marks), we observed strong depletions of H1 xxChIP epitope exposure, consistent with our previous analyses above. Interestingly, the profiles around centers of H3K36me3 domains (the mark of gene bodies of active genes) revealed less difference between xChIP and xxChIP epitope exposure, compared to the other shown histone modifications. Perhaps, this is because H3K36me3-enriched domains are wider than promoter/enhancer marks and less focused on their ChIP-seq peak summits. However, H3K36me3-domains did reveal a difference between H1.2 and H1.5 distribution, with enrichment of H1.2 and depletion of H1.5.

### Influence of H1.2/H1.5 enrichment on the nucleosome repeat length (NRL)

**Fig. 8:**
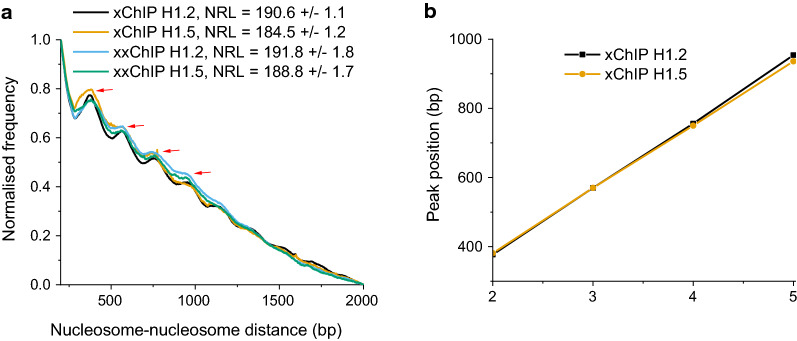
Nucleosome repeat length (NRL) calculated inside chromatin domains enriched with H1.2 and H1.5 based on xChIP and xxChIP, as indicated in the figure. **a** Normalized frequency distribution of nucleosome–nucleosome distances. **b** Linear fit of the peak summit positions from the left panel. The slope of the fit line gives the NRL

NRL is defined as the average distance (bp) between the dyad axes of adjacent nucleosomes and is traditionally used as an integrative parameter characterizing local nucleosome packing. Previous publications suggest that NRL is different near binding sites of transcription factors [42] and affected by the presence of linker histones, although the role of different H1 variants is not clear [43]. Figure 8 presents normalized calculations of the NRL in regions enriched for the H1 variants, using the NucTools algorithm [27]. In the case of xxChIP, NRL was similar for H1.2 and H1.5 (191.8 bp and 188.8 bp, respectively). In the case of xChIP, the difference between H1.2 and H1.5 was slightly larger (190.6 bp and 184.5 bp, respectively). These measurements suggest that chromatin regions enriched with either H1.2 or H1.5 may have different arrangements of nucleosomes.

### Interplay between linker histones and DNA methylation

**Fig. 9:**
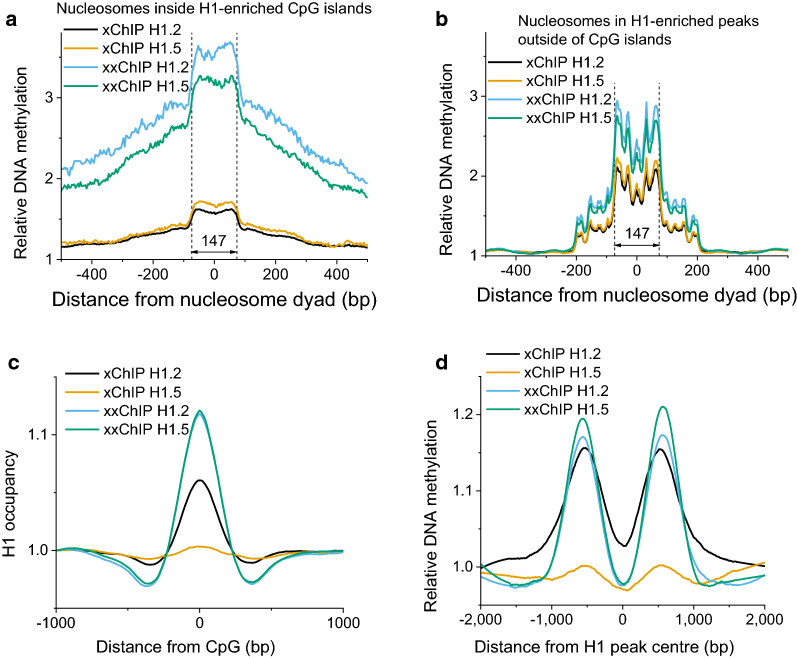
**a**, **b** Average profiles of DNA methylation aligned with respect to the centers of nucleosomes determined in HL-60/S4 cells using MNase-assisted xChIP-seq of histone H3 for nucleosomes inside peaks enriched with xChIP H1.2 (black) and xChIP H1.5 (orange), as well as xxChIP-seq of histone H1.2 (blue) and H1.5 (green). **a** Only nucleosomes inside H1-enriched peaks *inside* CpG islands are considered. **b** Only nucleosomes inside H1-enriched peaks *outside* CpG islands. **c** Average profiles of H1 epitope exposure around individual CpGs genome-wide. **d** Average profiles of DNA methylation around centers of H1-enriched peaks

To investigate the relationship between linker histones and DNA methylation, we performed whole genome bisulfite sequencing profiling in HL-60/S4 cells [30]. From our previous publications it is known that DNA methylation profiles around nucleosomes have well-defined patterns, which are significantly different depending on whether the nucleosome is located inside a CpG island or outside of CpG islands [38, 44]. Therefore, in the following analysis we take nucleosomes previously mapped using MNase-assisted H3 ChIP-seq in HL-60/S4 cells [31], and split them into two classes depending on their location inside or outside CpG islands. Furthermore, we narrow down this dataset to take into account only those nucleosomes which are located inside genomic locations enriched with one of four H1-related signals determined here (xChIP H1.2 and H.5 and xxChIP H1.2 and H1.5). Figure 9a, b demonstrates the average DNA methylation profiles calculated around the centers (dyads) of nucleosomes split into these 8 classes.

Figure 9a shows the DNA methylation profiles around nucleosomes inside CpG islands. These profiles are consistent with the idea that CpG islands are in general depleted of nucleosomes, but those few nucleosomes that appear in CpG islands are strongly associated with DNA methylation. These profiles are very different between xChIP and xxChIP, consistent with our previous calculations in Figs. 4 and 6d which show the largest differences between xChIP and xxChIP among all genomic features. DNA methylation profiles around nucleosomes outside of CpG islands are not so dramatically different between xChIP and xxChIP. Quantitatively, average DNA methylation profiles around all nucleosomes showed that DNA methylation was in general higher for xxChIP H1-enriched nucleosomes than xChIP H1-enriched nucleosomes. This can be explained by the increased CpG density in/near xxChIP DNA fragments, with both H1.2 and H1.5 xxChIP showing strong enrichment near CpGs (Fig. 9c). On the other hand, when we considered genome domains enriched with linker histones based on MUSIC peak calling, DNA methylation was depleted in the centers of the H1-enriched peaks and increased at a distance about 500 bp from the centers of the peaks (Fig. 9d); the latter effect was consistent with the GC content signatures of these peaks (Additional file 1: Figure S3). Thus, chromatin regions differentially enriched with H1.2/H1.5 in xChIP/xxChIP are characterized by distinct DNA methylation profiles which may reflect differences in nucleosome packing.

## Discussion

We used a combination of a newly introduced xxChIP-seq method (23), together with traditional xChIP-seq (Fig. 1) to study the differential distribution and epitope “exposure” of linker histone variants H1.2 and H1.5 in the human leukemia cell line HL-60/S4. In xxChIP-seq, the first fixation stabilizes in situ chromatin higher-order structure, which is destroyed by the sonication step in xChIP. The unique feature of xxChIP-seq, compared to xChIP-seq, is that the second formaldehyde fixation stabilizes specifically bound antibody during washing, sonication and processing of antibody-bound fragments. Occlusion of H1 epitope signals at a particular chromosomal site (i.e., epitope “hidden” in xxChIP, but “exposed” in xChIP) suggests the existence of higher-order chromatin structure at that particular site, but does not explain what this structure is. It is important to point out that “chromatin higher-order structure” exists at many scales. For example, “chromomeres” (i.e., formaldehyde fixed punctate chromatin structures (12), shown in Fig. 2) may contain ~ 10^3^ or more nucleosomes (corresponding to ~ 2 × 10^5^ or more bp). The peaks of H1 enrichment that we identify by xxChIP-seq are much smaller (~ 10^4^ or less bp; see Additional file 1: Figure S2). So, a chromomere might contain ~ 20 or more of the structural regions identified by xxChIP-seq.

Our results indicate that H1.2 xChIP-seq and H1.5 xChIP-seq signals are positively correlated; H1.2 xxChIP-seq and H1.5 xxChIP-seq signals are also positively correlated; but H1 xChIP-seq versus H1 xxChIP signals are negatively correlated (Fig. 3a, b). These differences between xChIP and xxChIP are visible along all human chromosomes (Fig. 3a and Additional file 1: Figure S4). While xChIP H1.2 and H1.5 signals are positively correlated genome wide, we identified several thousand regions where H1.2 dominates over H1.5 (or vice versa) at a “microdomain” scale, comprising a swing for a few nucleosomes (Fig. 3c, d). Interestingly, many of these are inside CpG islands and functional regulatory regions, with about 40% of them overlapping with promoters enriched for genes encoding ATP-binding proteins. Such “swings” between H1.2 and H1.5 in small microdomains comprising few nucleosomes may correspond to “clutches” of nucleosomes reported recently [45]. It is worth noting that both xChIP and xxChIP report cell population-averaged data, whereas individual cells may experience intrinsic stochasticity of chromatin organization [46]. Thus, it is remarkable that we are able to observe few thousands of clutches of nucleosomes with mutually exclusive H1.2 or H1.5 at functionally important regions, but it could be that individual cells have even more such regions which became cancelled out after averaging over the large population of cells in the bulk experiment.

It is important to emphasize that all types of transcriptionally active or “open” regions such as CpG islands were more enriched by H1 xChIP than xxChIP, whereas Alu and L1 repeats were more enriched with H1 xxChIP (Fig. 4). Epichromatin regions (i.e., “surface chromatin”), operationally defined by the binding of the bivalent mAb PL2-6 (12,23,24,34), were depleted within enriched H1 xxChIP peaks, but enriched within H1 xChIP peaks (Fig. 4), suggesting that epichromatin domains have unique chromatin structures with “hidden” H1 epitopes.

Several chromatin-bound proteins exhibited clear preferences for “association” with regions enriched in different histone H1 variants (Fig. 5). In particular, regions associated with the cohesin subunits SMC3 and STAG1, as well as the proteins REST and RNA Pol II, were among those where the differences between H1.2 and H1.5 were more pronounced in xChIP-seq. The “non-classical pioneer factor” PU.1 [41], which is important for the fate of HL60/S4 cells, showed the opposite tendencies in xChIP and xxChIP average profiles, suggesting different types of PU.1-sensitive exposure of linker histones. Interestingly, the abundant CTCF-binding sites did not show preferences for the studied H1 variants. Our conception is that H1 epitope exposure correlates well with H1 “occupancy”, when considering xChIP-seq analysis, but less well when analyzing xxChIP-seq, because of the generation of “hidden” epitopes.

The investigation of the relationship between active/inactive gene promoters and H1 binding revealed an unexpected observation. The depletion of H1.2 and H1.5 determined by xChIP is quite narrowly localized within ~ 200 bp of the active (but not inactive) TSS. This is consistent with the recently reported H1 xChIP-seq profiles in Drosophila embryonic development [47]. On the other hand, in the case of xxChIP-seq, the depleted region is much broader, covering more than 2 kb from TSS (Fig. 6). The latter effect for xxChIP can be observed both for active and inactive TSS. Comparing genomic regions enriched with different modifications of core histones (Fig. 7), we found that xChIP (but not xxChIP) detects large differences between H1.2 versus H1.5. In general, the level of H1.2 was higher than H1.5 for regions enriched with “active” post-translational modifications of core histones. The breadth of the “dip” around active TSS, as seen with xxChIP for anti-H1s, may be explained by the fact that active genes appear to associate with higher-order structures; e.g., “hubs” or “factories” [48]. Presumably, with xxChIP these complexes are better preserved, than in xChIP. We also cannot exclude that the second fixation in the xxChIP protocol might have preferentially “stiffened” the nucleosome-depleted regions surrounding the TSS, making them less susceptible to sonication, but such effect, if present, does not explain the widening of the “open” region in xxChIP in comparison with xChIP. Thus, the most likely explanation of the widening of this region in xxChIP is the incorporation of H1 within the complexes of non-histone proteins. Interestingly, the signature around active TSSs is also sharper for H1.2 than H1.5 (Fig. 6a), which may be important for the role of H1.5 in binding over splice sites and regulating alternative splicing, as reported recently [49].

Our analysis also indicates some changes of nucleosome packing, characterized by an NRL change from 190 to 184 bp for the areas enriched with H1.2 versus H1.5, respectively (Fig. 8). This suggests that different histone H1 variants can influence the structure of the nucleosome arrays, which may be accomplished by any of several different mechanisms, including a change in H1-nucleosome stoichiometry [43, 50]. Since we also observed that H1.2- versus H1.5-enriched regions are differentially methylated (Fig. 9), it appears that enrichment of different linker histone variants can be an important determinant of the physical packing and activity of chromatin microdomains at the scale of several nucleosomes. These findings are consistent with the recently reported cooperativity between H1 histones and DNA methylation in repressing transposable elements [51] and in establishing heterochromatin [52].

Given the acknowledged high in vivo mobility and structural redundancy of different H1 variants, the presently described localizations and nuclear element enrichments of H1.2 and H1.5 cannot be regarded as universal to other mammalian cells. Since undifferentiated HL-60/S4 cells are the object of interest within this study, the next logical step could be to examine H1 xChIP-seq versus xxChIP-seq in the differentiated granulocyte and macrophage cell states [31], to identify how differential gene expression has influenced H1 localization. Furthermore, the method reported here, comparing xChIP-seq versus xxChIP-seq to ascertain whether a specific chromatin protein epitope is “hidden” due to chromatin higher-order structure, has a general applicability to other cell types and other chromatin proteins.

## Supplementary information

**Additional file 1:** Additional Figures.

**Additional file 2:** Additional Table S1.

**Additional file 3:** Additional Table S2.

## Data Availability

xChIP and xxChIP datasets reported in this study are available in the GEO database (GSE136264).
