# Supplementary material for: Linker histone epitopes are hidden by in situ higher-order chromatin structure
Source: Epigenetics Chromatin. 2020 Jun 6;13:26. doi: 10.1186/s13072-020-00345-9 (PMC7276084; doi:10.1186/s13072-020-00345-9)
Supplement: Supplementary file 1 — Additional file 1: Additional Figures. [file 13072_2020_345_MOESM1_ESM.pdf]

Additional File 1

Linker histone epitopes are hidden by in situ higher-order chromatin structure

Vladimir B Teif[1,*], Travis J Gould[2], Christopher T Clarkson[1], Logan Boyd[2,3], Enoch B Antwi[4,5], Naveed Ishaque[6], Ada L Olins[7] & Donald E Olins[7,*]

[1] School of Life Sciences, University of Essex, Wivenhoe Park, Colchester, CO4 3SQ, UK

[2] Department of Physics & Astronomy, Bates College, Lewiston, ME, USA

[3] Current address: StarBird Technologies, LLC, Brunswick, ME

[4] Division of Theoretical Bioinformatics, German Cancer Research Center (DKFZ), Heidelberg, Germany.

[5] Molecular and Cellular Engineering, Centre for Biological Signalling Studies, University of Freiburg, Germany.

[6] Digital Health Center, Berlin Institute of Health (BIH) and Charité Universitätsmedizin, Berlin, Germany

[7] Department of Pharmaceutical Sciences, College of Pharmacy, University of New England, 716 Stevens Avenue, Portland, ME 04103, USA.

[*] Correspondence should be addressed to Donald E Olins (dolins@une.edu) or Vladimir B Teif (vteif@essex.ac.uk).

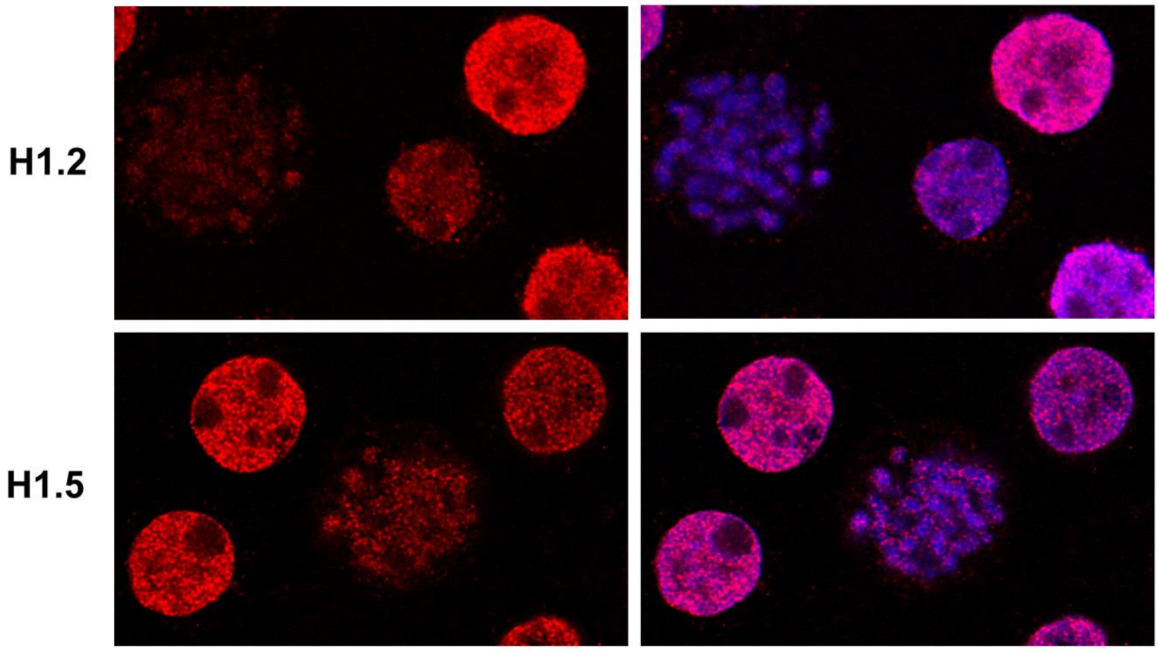

Figure S1. Confocal imaging of interphase and mitotic undifferentiated HL-60/S4 cells stained with anti-H1.2 (top row) including DAPI (right column) or with anti-H1.5 (bottom row) including DAPI (right column). Note that anti-H1.2 stains the mitotic chromosomes weaker than does anti-H1.5; but the staining of interphase nuclei are comparable.

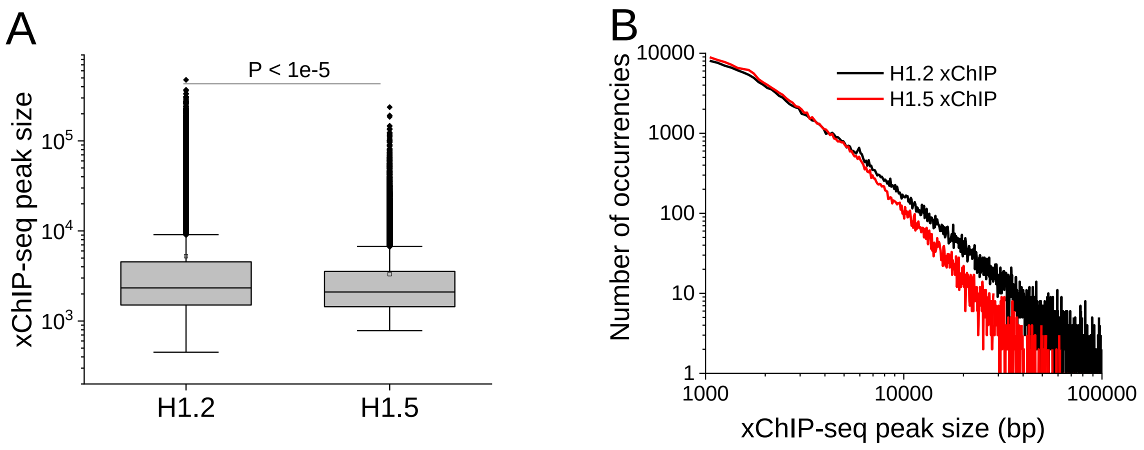

Figure S2. Analysis of the size (bp) distribution of chromatin peaks enriched with anti-H1.2 and anti-H1.5 based on xChIP-seq. Peak calling was performed using MUSIC.

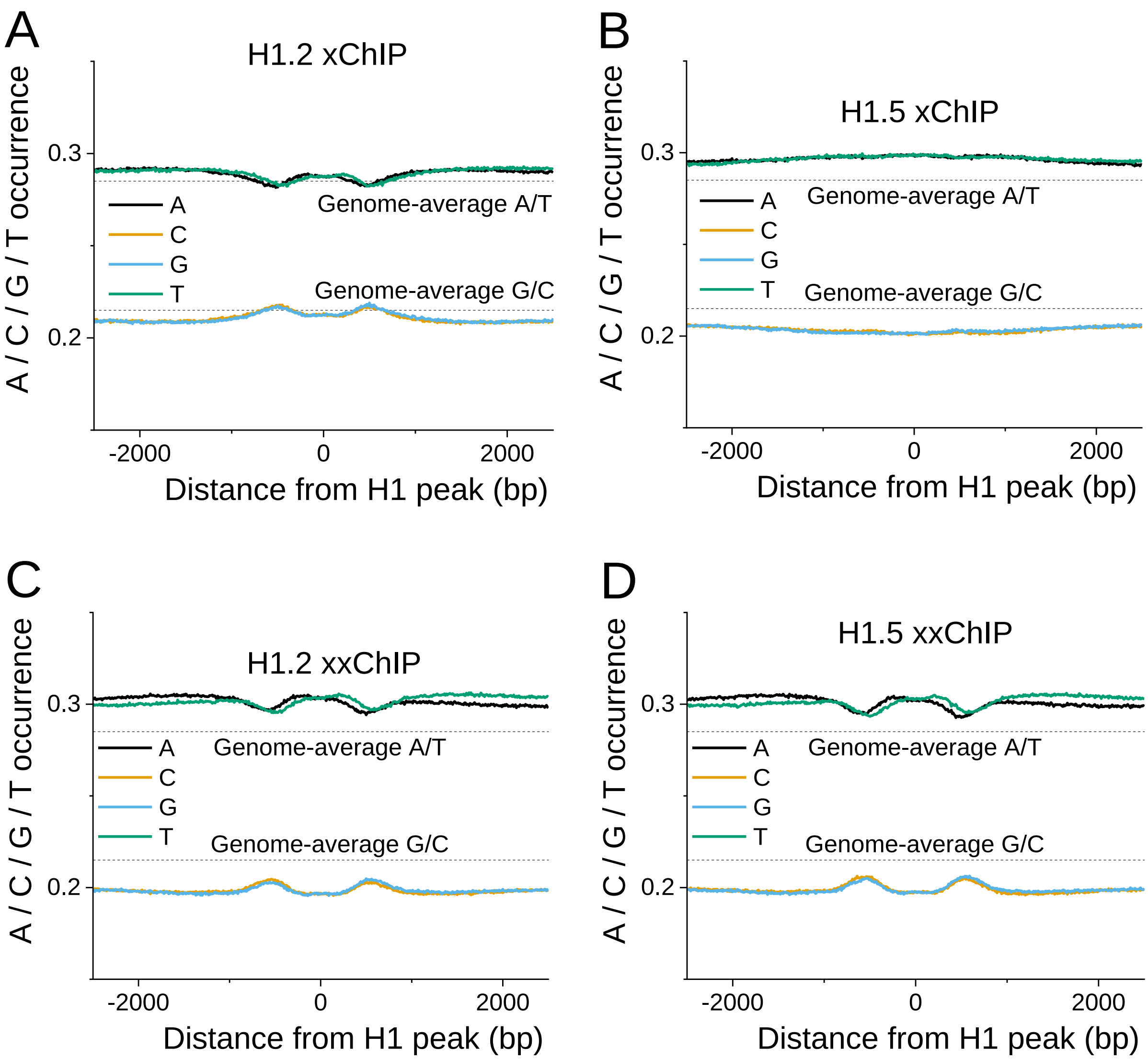

Figure S3. Nucleotide content around MUSIC-called peaks enriched for H1 variants.

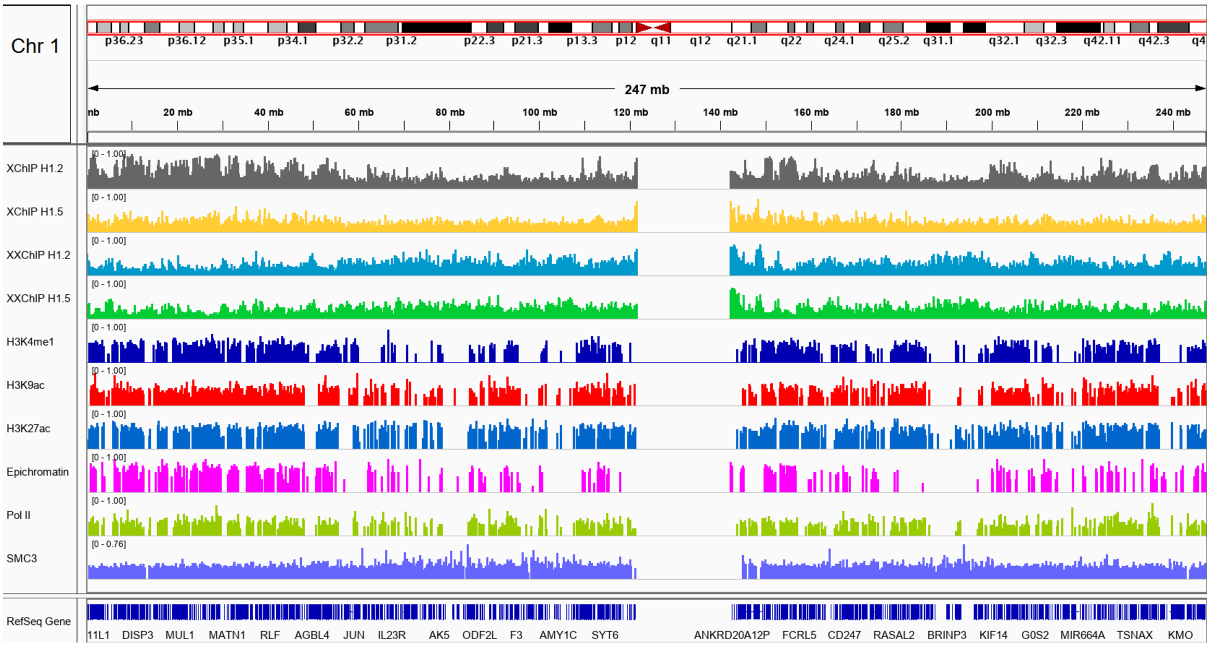

Figure S4. Large-scale (low resolution) comparisons between xChIP and xxChIP sequencing strategies for all the human chromosomes. H1 epitope “exposure” peak densities are measured by scanning with a window of 1000 base pairs: for xChIP H1.2 (black), xChIP H1.5 (orange), xxChIP H1.2 (blue) and xxChIP H1.5 (green). Also indicated are cytogenetic road marks, chromosome coordinates (mb), the epichromatin track (Olins et al., 2014), and tracks showing the density of peaks enriched with H3K4me1, H3K9ac, H3K27ac, Pol II and SMC3.

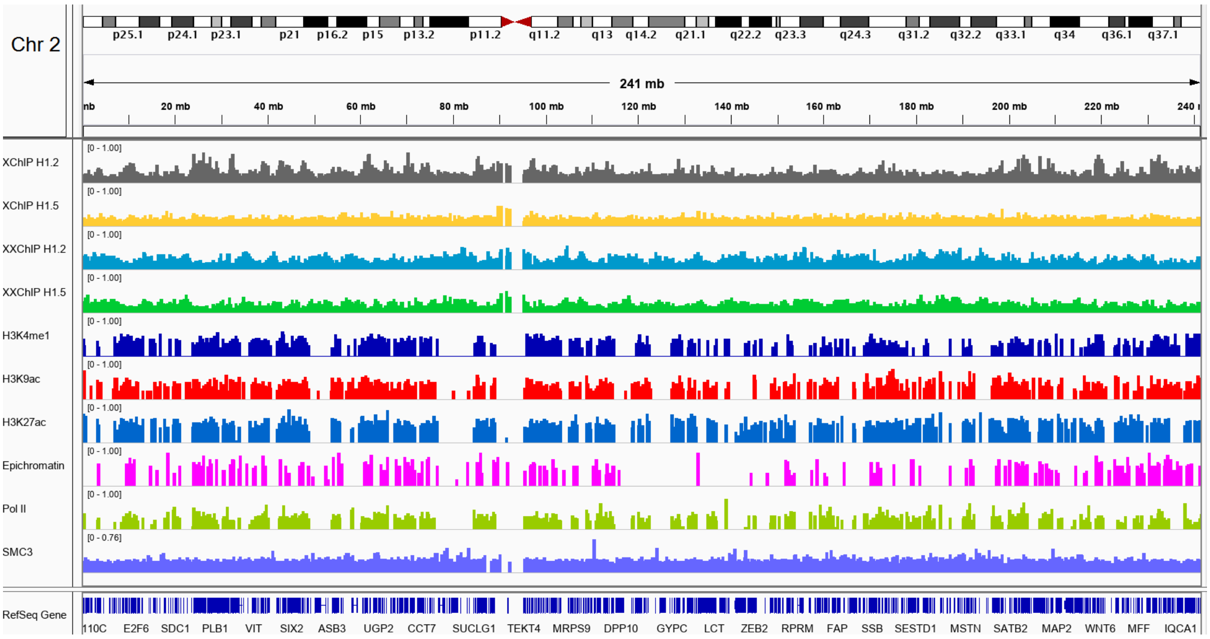

Figure S4. Large-scale (low resolution) comparisons between xChIP and xxChIP sequencing strategies for all the human chromosomes. H1 epitope “exposure” peak densities are measured by scanning with a window of 1000 base pairs: for xChIP H1.2 (black), xChIP H1.5 (orange), xxChIP H1.2 (blue) and xxChIP H1.5 (green). Also indicated are cytogenetic road marks, chromosome coordinates (mb), the epichromatin track (Olins et al., 2014), and tracks showing the density of peaks enriched with H3K4me1, H3K9ac, H3K27ac, Pol II and SMC3.

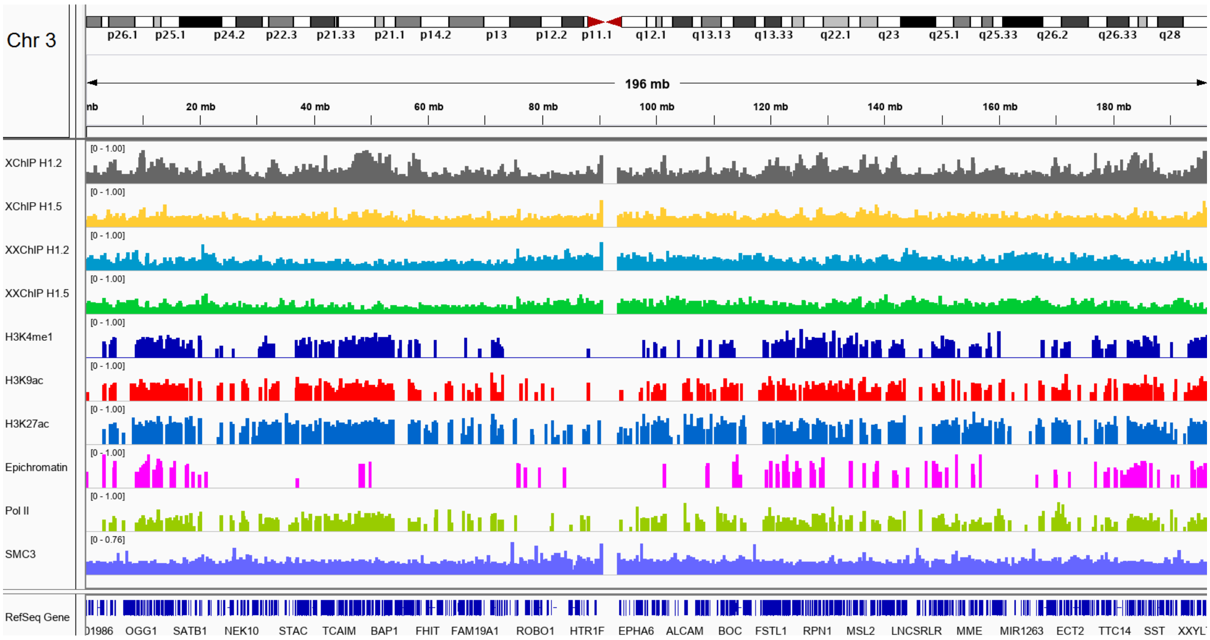

Figure S4. Large-scale (low resolution) comparisons between xChIP and xxChIP sequencing strategies for all the human chromosomes. H1 epitope “exposure” peak densities are measured by scanning with a window of 1000 base pairs: for xChIP H1.2 (black), xChIP H1.5 (orange), xxChIP H1.2 (blue) and xxChIP H1.5 (green). Also indicated are cytogenetic road marks, chromosome coordinates (mb), the epichromatin track (Olins et al., 2014), and tracks showing the density of peaks enriched with H3K4me1, H3K9ac, H3K27ac, Pol II and SMC3.

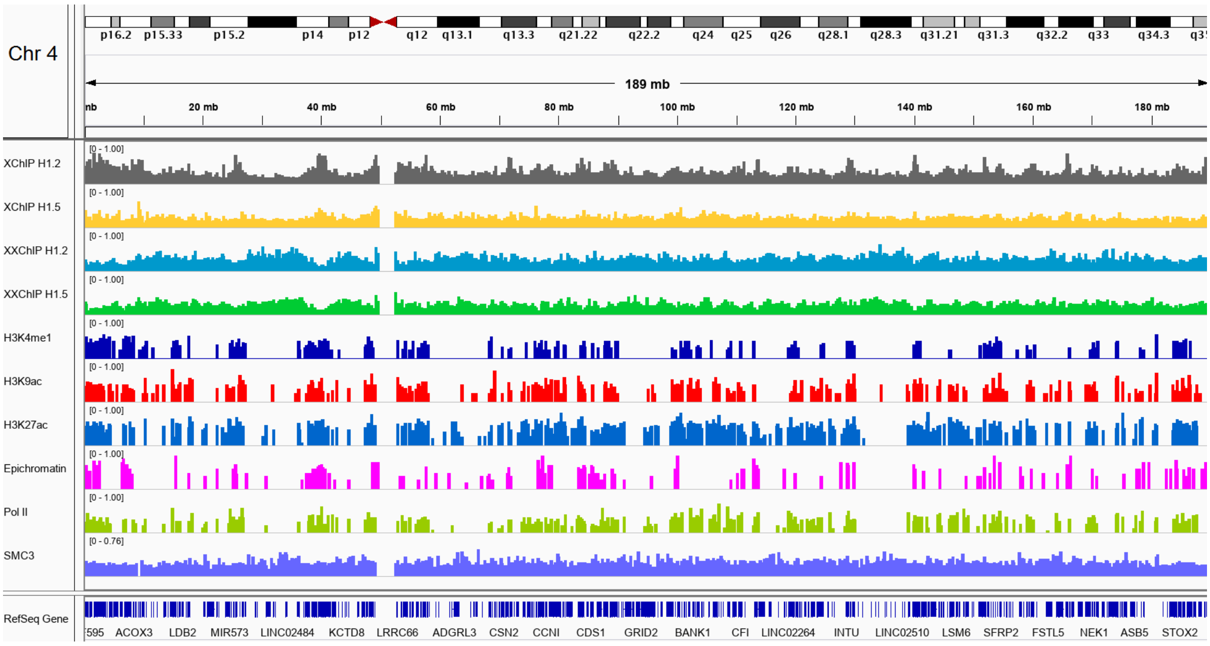

Figure S4. Large-scale (low resolution) comparisons between xChIP and xxChIP sequencing strategies for all the human chromosomes. H1 epitope “exposure” peak densities are measured by scanning with a window of 1000 base pairs: for xChIP H1.2 (black), xChIP H1.5 (orange), xxChIP H1.2 (blue) and xxChIP H1.5 (green). Also indicated are cytogenetic road marks, chromosome coordinates (mb), the epichromatin track (Olins et al., 2014), and tracks showing the density of peaks enriched with H3K4me1, H3K9ac, H3K27ac, Pol II and SMC3.

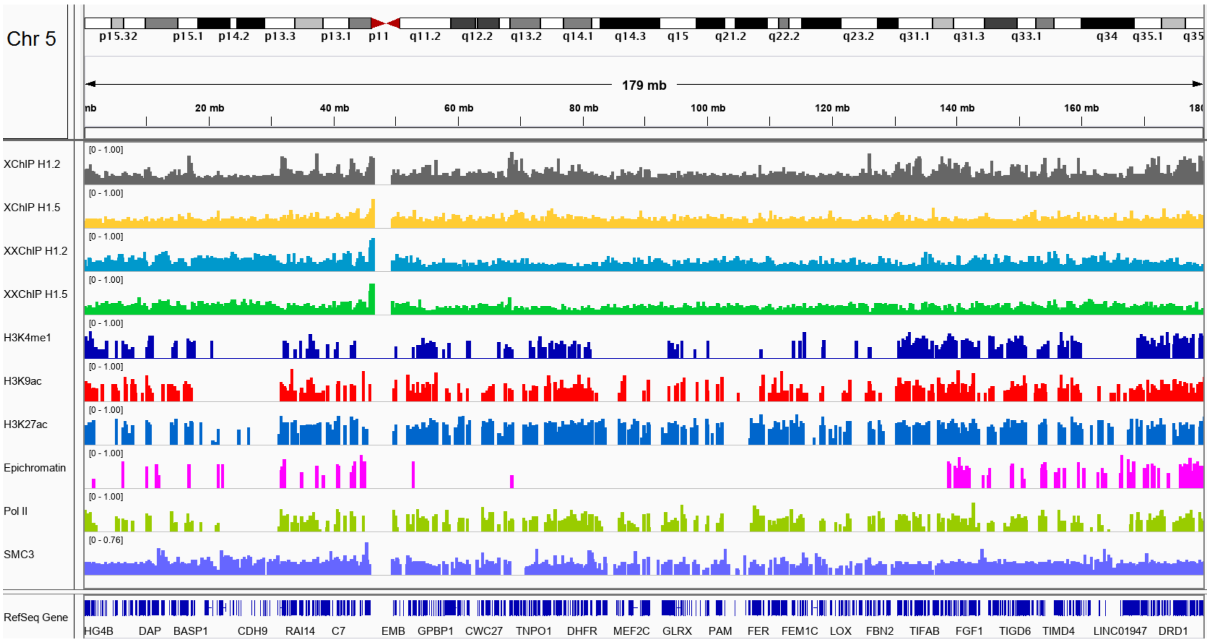

Figure S4. Large-scale (low resolution) comparisons between xChIP and xxChIP sequencing strategies for all the human chromosomes. H1 epitope “exposure” peak densities are measured by scanning with a window of 1000 base pairs: for xChIP H1.2 (black), xChIP H1.5 (orange), xxChIP H1.2 (blue) and xxChIP H1.5 (green). Also indicated are cytogenetic road marks, chromosome coordinates (mb), the epichromatin track (Olins et al., 2014), and tracks showing the density of peaks enriched with H3K4me1, H3K9ac, H3K27ac, Pol II and SMC3.

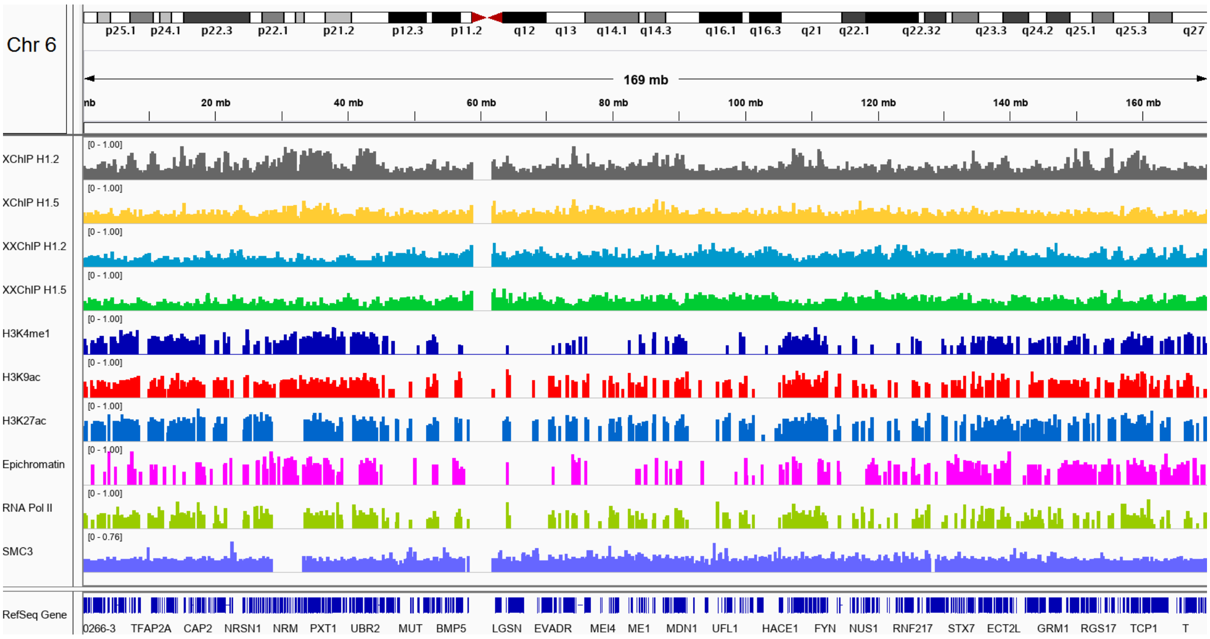

Figure S4. Large-scale (low resolution) comparisons between xChIP and xxChIP sequencing strategies for all the human chromosomes. H1 epitope “exposure” peak densities are measured by scanning with a window of 1000 base pairs: for xChIP H1.2 (black), xChIP H1.5 (orange), xxChIP H1.2 (blue) and xxChIP H1.5 (green). Also indicated are cytogenetic road marks, chromosome coordinates (mb), the epichromatin track (Olins et al., 2014), and tracks showing the density of peaks enriched with H3K4me1, H3K9ac, H3K27ac, Pol II and SMC3.

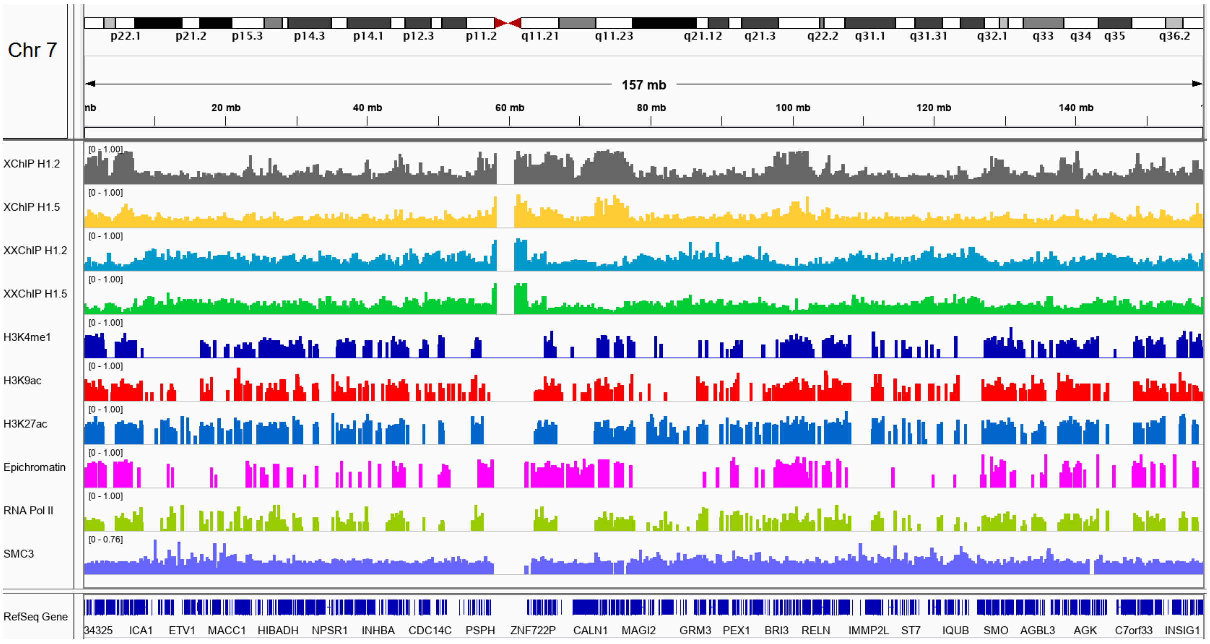

Figure S4. Large-scale (low resolution) comparisons between xChIP and xxChIP sequencing strategies for all the human chromosomes. H1 epitope “exposure” peak densities are measured by scanning with a window of 1000 base pairs: for xChIP H1.2 (black), xChIP H1.5 (orange), xxChIP H1.2 (blue) and xxChIP H1.5 (green). Also indicated are cytogenetic road marks, chromosome coordinates (mb), the epichromatin track (Olins et al., 2014), and tracks showing the density of peaks enriched with H3K4me1, H3K9ac, H3K27ac, Pol II and SMC3.

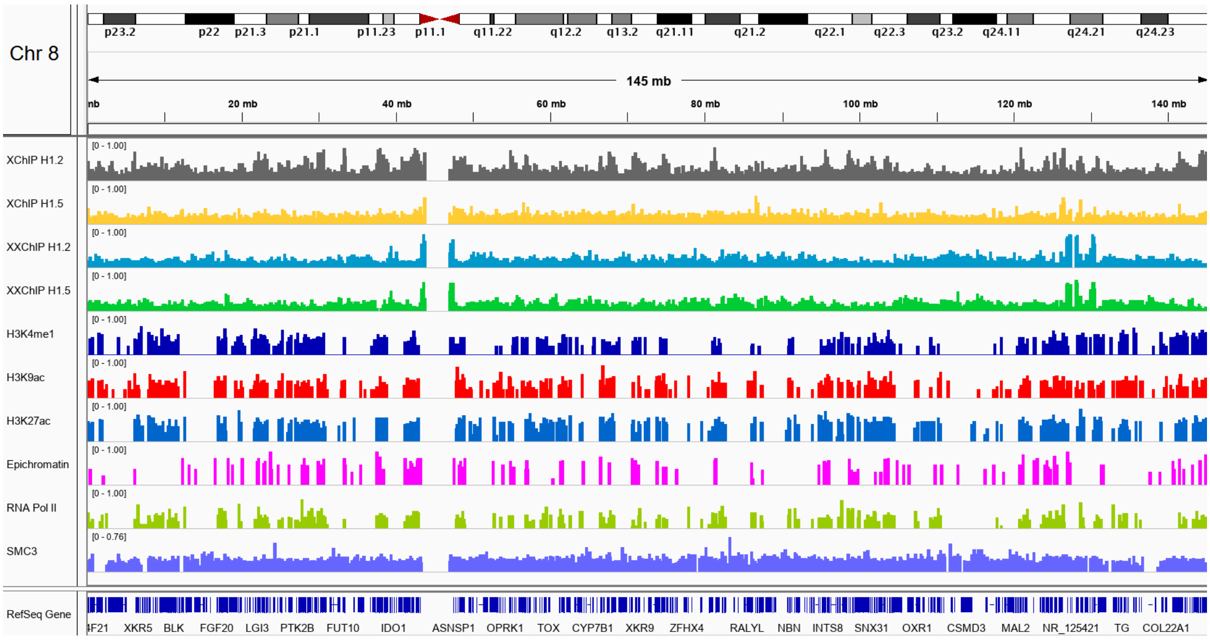

Figure S4. Large-scale (low resolution) comparisons between xChIP and xxChIP sequencing strategies for all the human chromosomes. H1 epitope “exposure” peak densities are measured by scanning with a window of 1000 base pairs: for xChIP H1.2 (black), xChIP H1.5 (orange), xxChIP H1.2 (blue) and xxChIP H1.5 (green). Also indicated are cytogenetic road marks, chromosome coordinates (mb), the epichromatin track (Olins et al., 2014), and tracks showing the density of peaks enriched with H3K4me1, H3K9ac, H3K27ac, Pol II and SMC3.

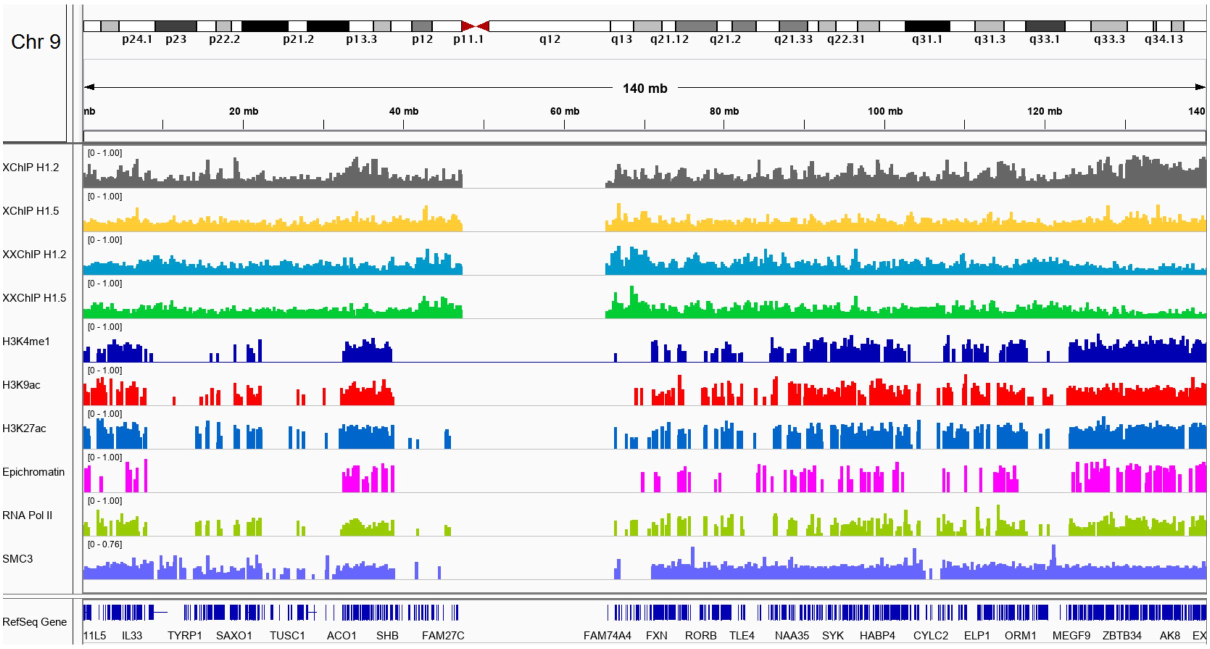

Figure S4. Large-scale (low resolution) comparisons between xChIP and xxChIP sequencing strategies for all the human chromosomes. H1 epitope “exposure” peak densities are measured by scanning with a window of 1000 base pairs: for xChIP H1.2 (black), xChIP H1.5 (orange), xxChIP H1.2 (blue) and xxChIP H1.5 (green). Also indicated are cytogenetic road marks, chromosome coordinates (mb), the epichromatin track (Olins et al., 2014), and tracks showing the density of peaks enriched with H3K4me1, H3K9ac, H3K27ac, Pol II and SMC3.

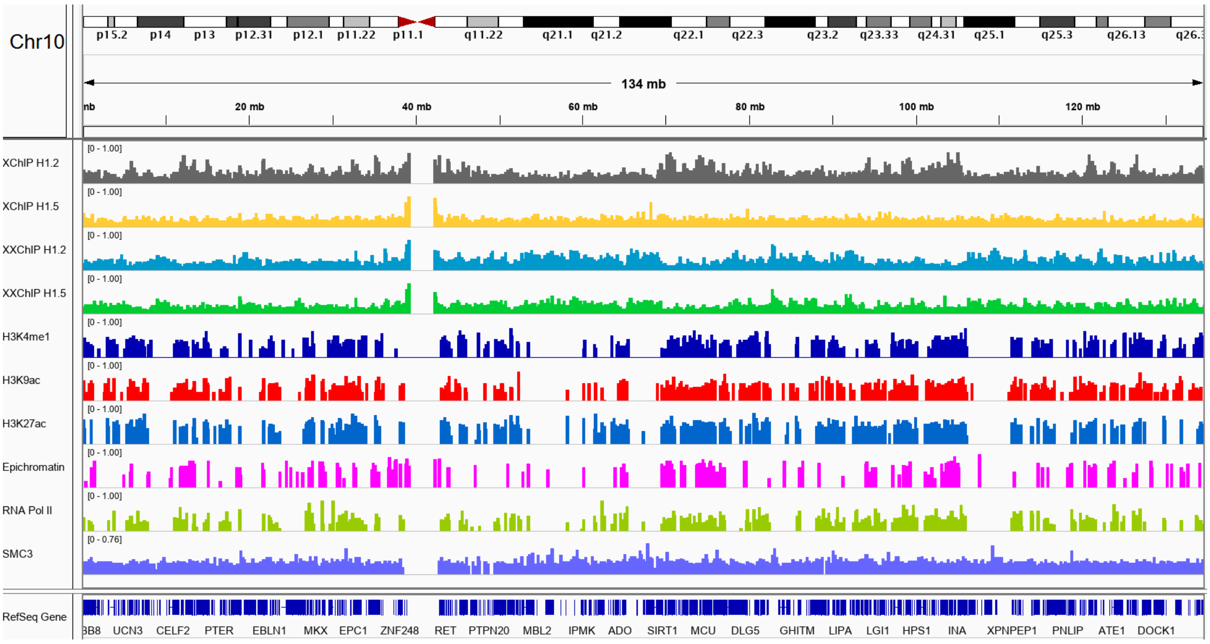

Figure S4. Large-scale (low resolution) comparisons between xChIP and xxChIP sequencing strategies for all the human chromosomes. H1 epitope “exposure” peak densities are measured by scanning with a window of 1000 base pairs: for xChIP H1.2 (black), xChIP H1.5 (orange), xxChIP H1.2 (blue) and xxChIP H1.5 (green). Also indicated are cytogenetic road marks, chromosome coordinates (mb), the epichromatin track (Olins et al., 2014), and tracks showing the density of peaks enriched with H3K4me1, H3K9ac, H3K27ac, Pol II and SMC3.

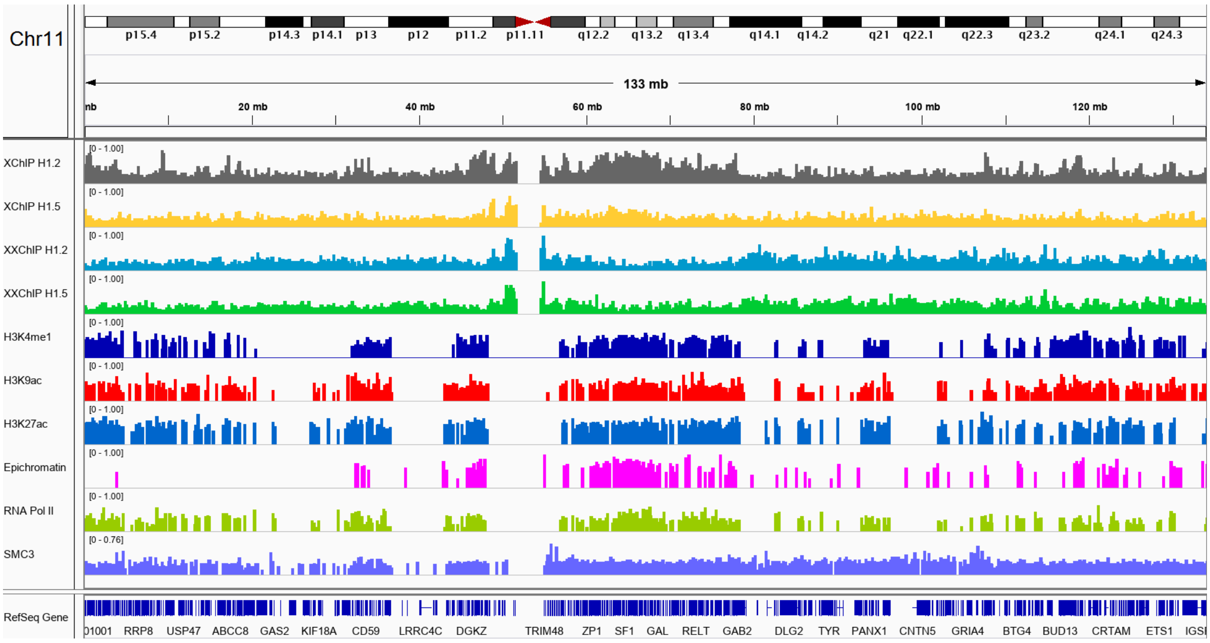

Figure S4. Large-scale (low resolution) comparisons between xChIP and xxChIP sequencing strategies for all the human chromosomes. H1 epitope “exposure” peak densities are measured by scanning with a window of 1000 base pairs: for xChIP H1.2 (black), xChIP H1.5 (orange), xxChIP H1.2 (blue) and xxChIP H1.5 (green). Also indicated are cytogenetic road marks, chromosome coordinates (mb), the epichromatin track (Olins et al., 2014), and tracks showing the density of peaks enriched with H3K4me1, H3K9ac, H3K27ac, Pol II and SMC3.

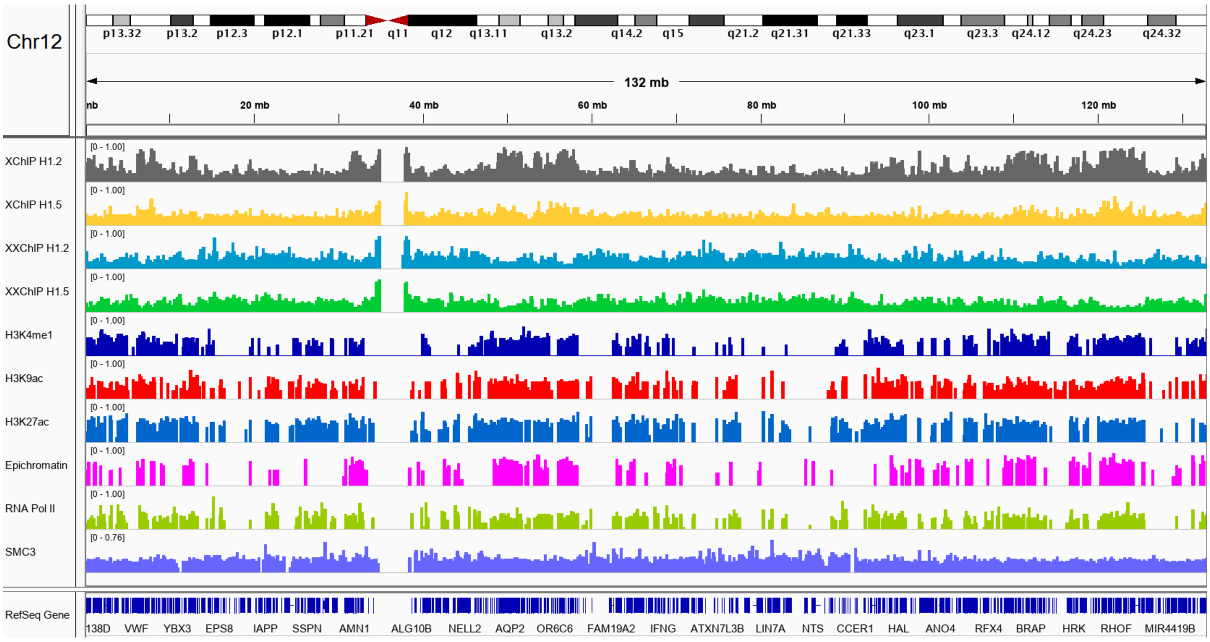

Figure S4. Large-scale (low resolution) comparisons between xChIP and xxChIP sequencing strategies for all the human chromosomes. H1 epitope "exposure" peak densities are measured by scanning with a window of 1000 base pairs: for xChIP H1.2 (black), xChIP H1.5 (orange), xxChIP H1.2 (blue) and xxChIP H1.5 (green). Also indicated are cytogenetic road marks, chromosome coordinates (mb), the epichromatin track (Olins et al., 2014), and tracks showing the density of peaks enriched with H3K4me1, H3K9ac, H3K27ac, Pol II and SMC3.

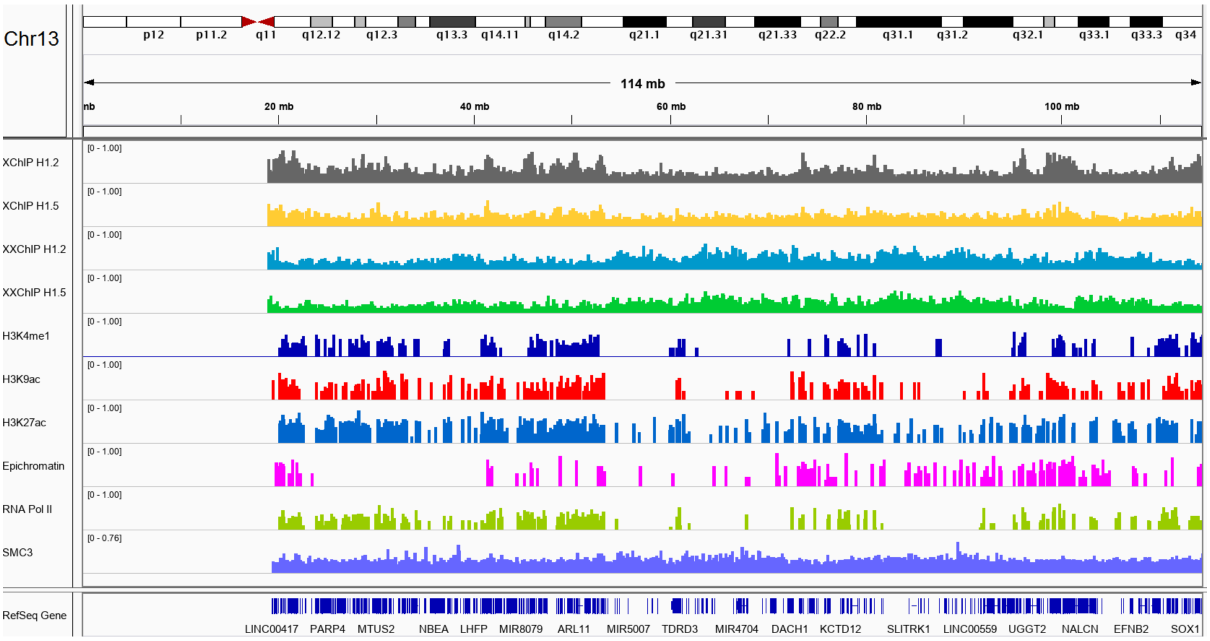

Figure S4. Large-scale (low resolution) comparisons between xChIP and xxChIP sequencing strategies for all the human chromosomes. H1 epitope “exposure” peak densities are measured by scanning with a window of 1000 base pairs: for xChIP H1.2 (black), xChIP H1.5 (orange), xxChIP H1.2 (blue) and xxChIP H1.5 (green). Also indicated are cytogenetic road marks, chromosome coordinates (mb), the epichromatin track (Olins et al., 2014), and tracks showing the density of peaks enriched with H3K4me1, H3K9ac, H3K27ac, Pol II and SMC3.

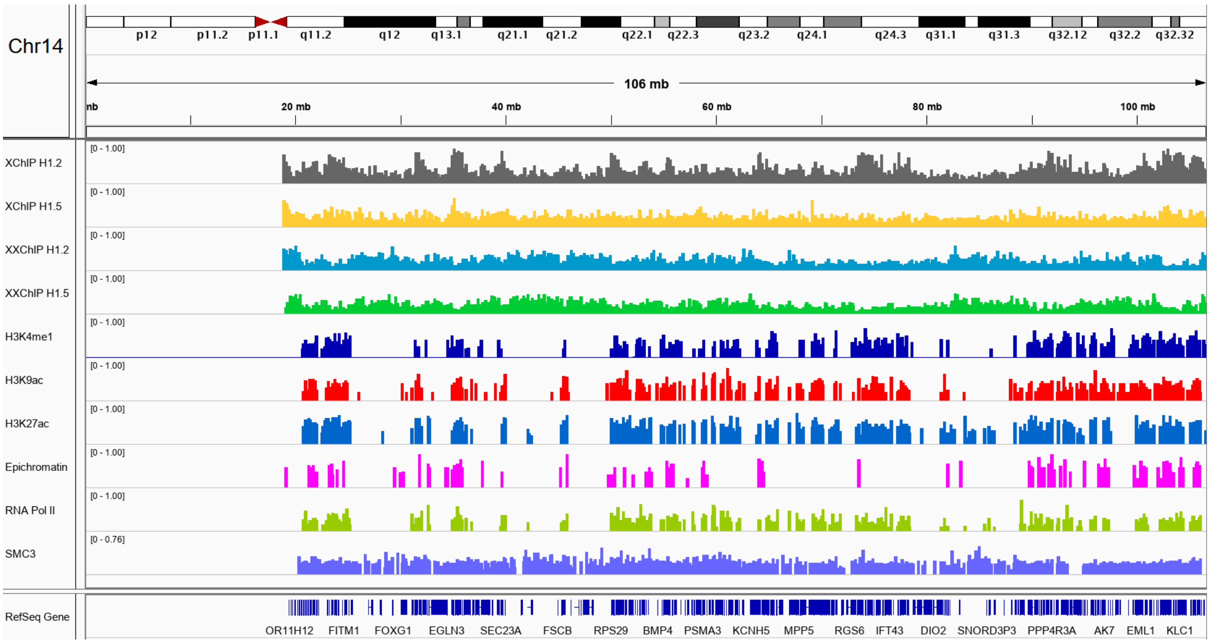

Figure S4. Large-scale (low resolution) comparisons between xChIP and xxChIP sequencing strategies for all the human chromosomes. H1 epitope "exposure" peak densities are measured by scanning with a window of 1000 base pairs: for xChIP H1.2 (black), xChIP H1.5 (orange), xxChIP H1.2 (blue) and xxChIP H1.5 (green). Also indicated are cytogenetic road marks, chromosome coordinates (mb), the epichromatin track (Olins et al., 2014), and tracks showing the density of peaks enriched with H3K4me1, H3K9ac, H3K27ac, Pol II and SMC3.

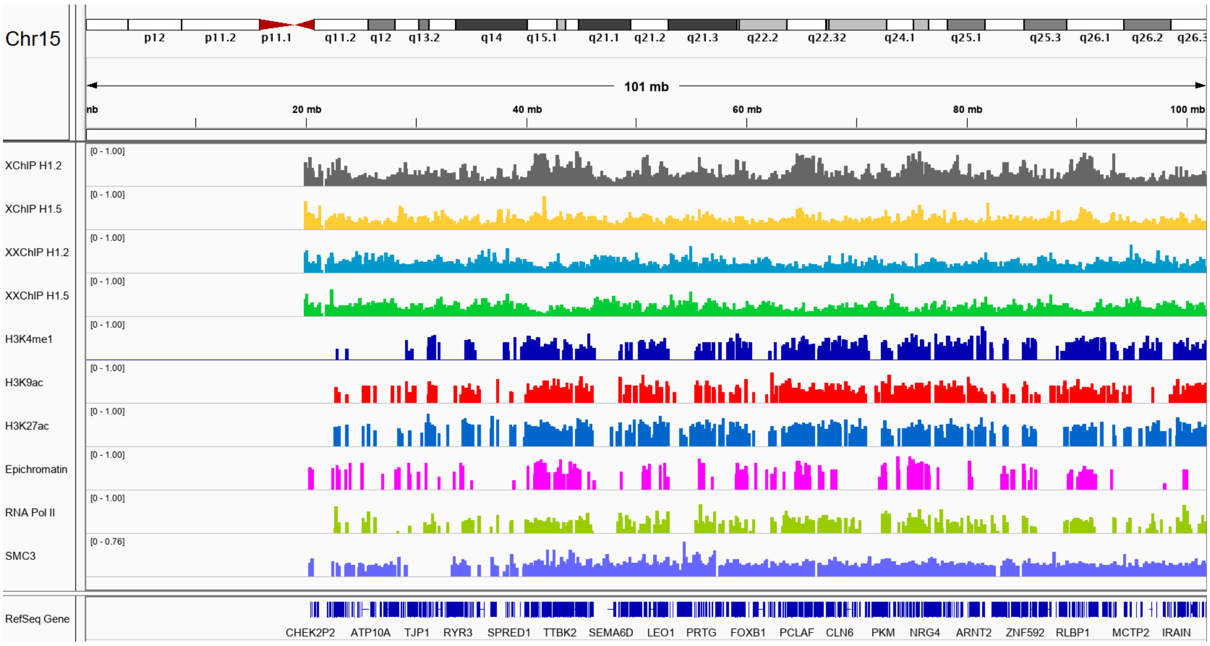

Figure S4. Large-scale (low resolution) comparisons between xChIP and xxChIP sequencing strategies for all the human chromosomes. H1 epitope “exposure” peak densities are measured by scanning with a window of 1000 base pairs: for xChIP H1.2 (black), xChIP H1.5 (orange), xxChIP H1.2 (blue) and xxChIP H1.5 (green). Also indicated are cytogenetic road marks, chromosome coordinates (mb), the epichromatin track (Olins et al., 2014), and tracks showing the density of peaks enriched with H3K4me1, H3K9ac, H3K27ac, Pol II and SMC3.

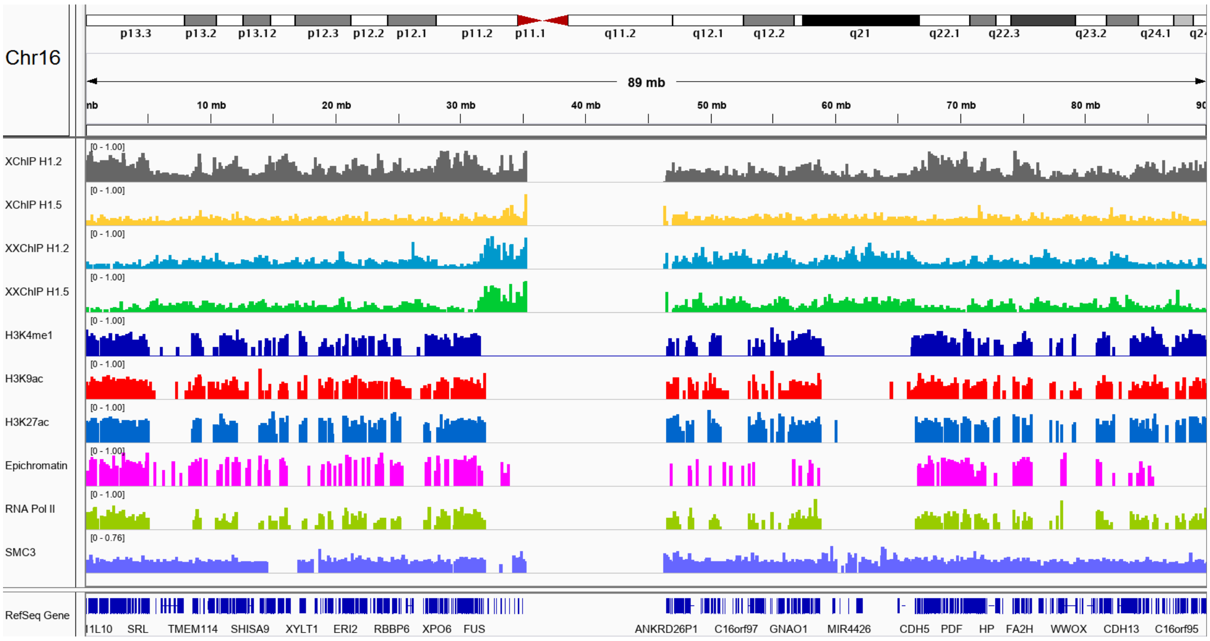

Figure S4. Large-scale (low resolution) comparisons between xChIP and xxChIP sequencing strategies for all the human chromosomes. H1 epitope “exposure” peak densities are measured by scanning with a window of 1000 base pairs: for xChIP H1.2 (black), xChIP H1.5 (orange), xxChIP H1.2 (blue) and xxChIP H1.5 (green). Also indicated are cytogenetic road marks, chromosome coordinates (mb), the epichromatin track (Olins et al., 2014), and tracks showing the density of peaks enriched with H3K4me1, H3K9ac, H3K27ac, Pol II and SMC3.

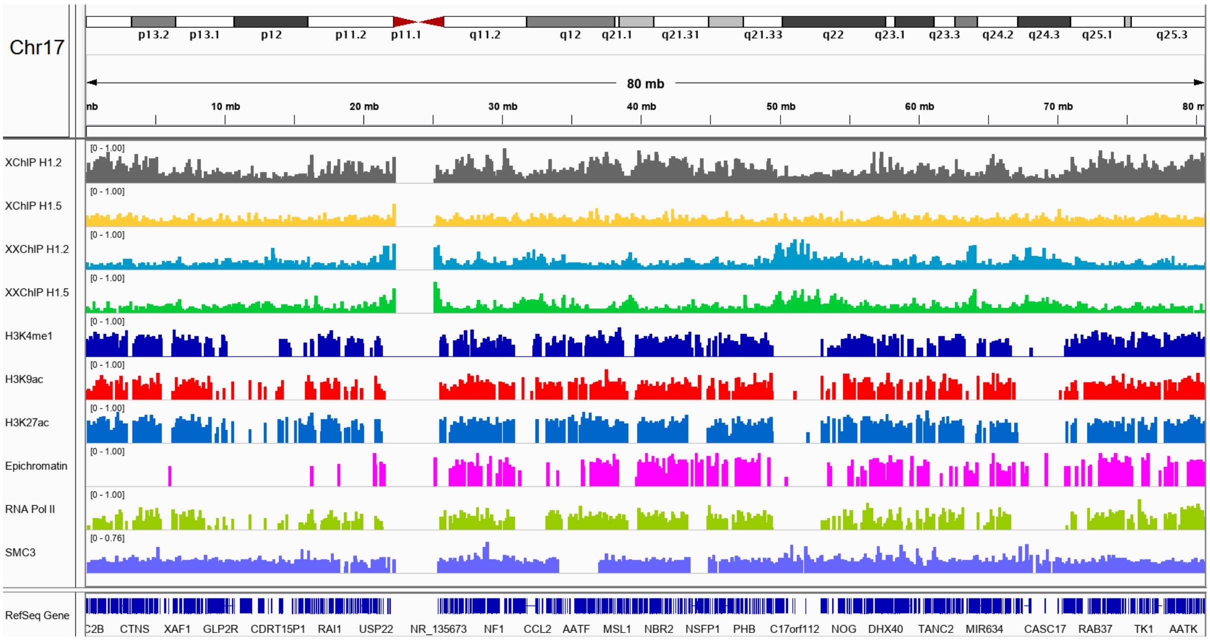

Figure S4. Large-scale (low resolution) comparisons between xChIP and xxChIP sequencing strategies for all the human chromosomes. H1 epitope “exposure” peak densities are measured by scanning with a window of 1000 base pairs: for xChIP H1.2 (black), xChIP H1.5 (orange), xxChIP H1.2 (blue) and xxChIP H1.5 (green). Also indicated are cytogenetic road marks, chromosome coordinates (mb), the epichromatin track (Olins et al., 2014), and tracks showing the density of peaks enriched with H3K4me1, H3K9ac, H3K27ac, Pol II and SMC3.

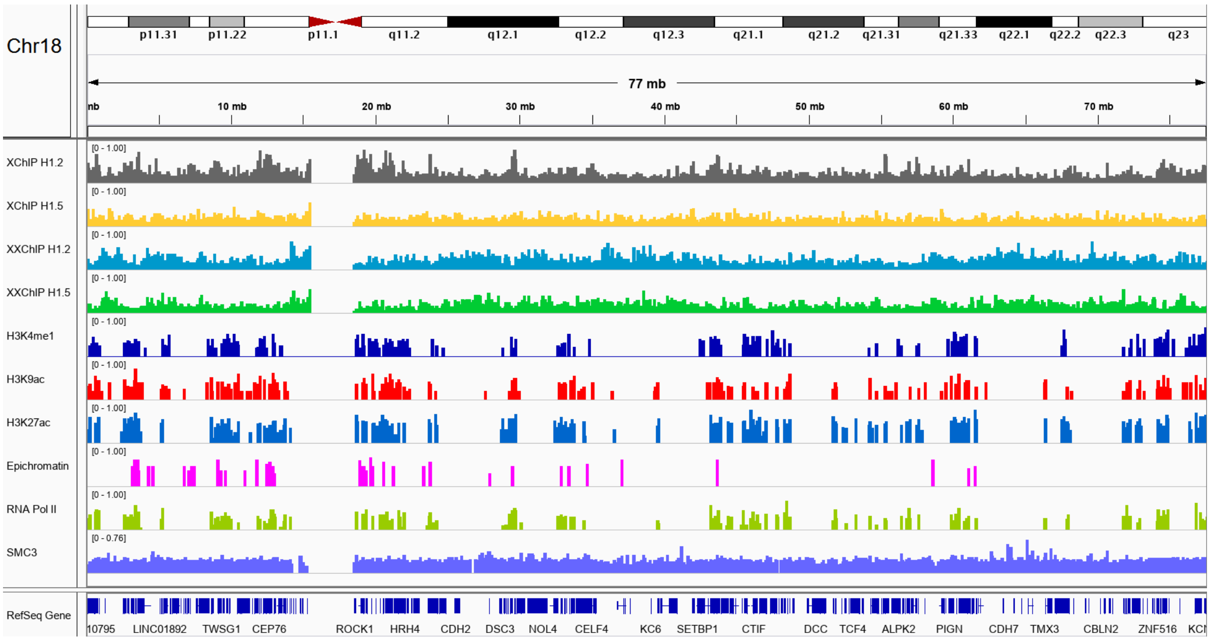

Figure S4. Large-scale (low resolution) comparisons between xChIP and xxChIP sequencing strategies for all the human chromosomes. H1 epitope "exposure" peak densities are measured by scanning with a window of 1000 base pairs: for xChIP H1.2 (black), xChIP H1.5 (orange), xxChIP H1.2 (blue) and xxChIP H1.5 (green). Also indicated are cytogenetic road marks, chromosome coordinates (mb), the epichromatin track (Olins et al., 2014), and tracks showing the density of peaks enriched with H3K4me1, H3K9ac, H3K27ac, Pol II and SMC3.

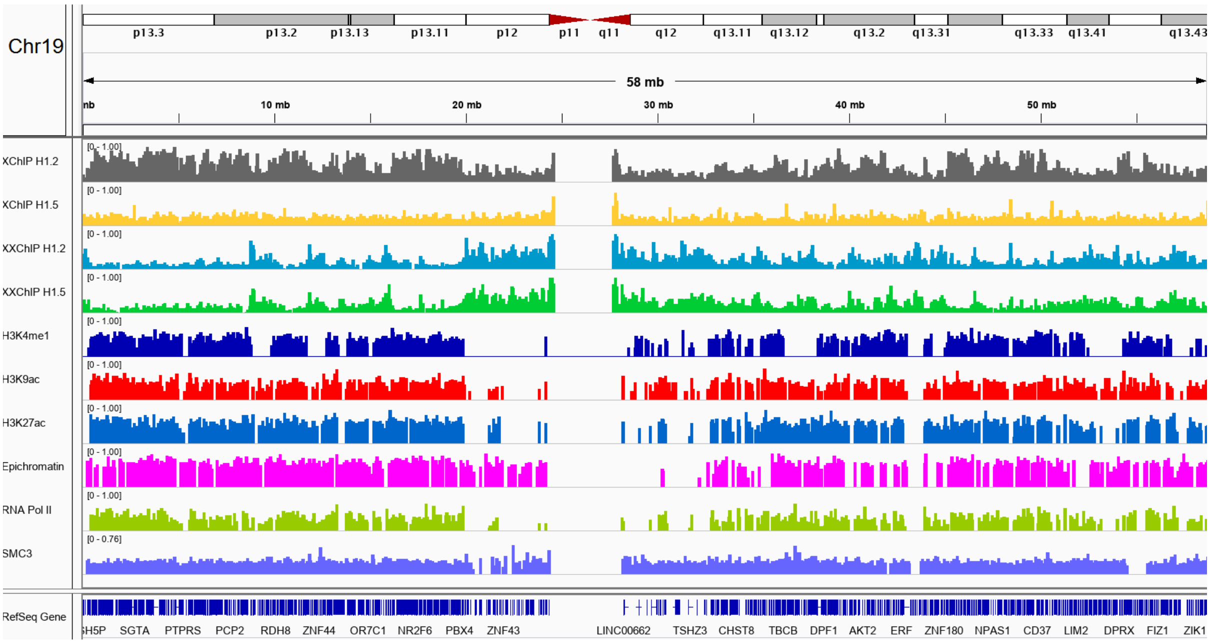

Figure S4. Large-scale (low resolution) comparisons between xChIP and xxChIP sequencing strategies for all the human chromosomes. H1 epitope “exposure” peak densities are measured by scanning with a window of 1000 base pairs: for xChIP H1.2 (black), xChIP H1.5 (orange), xxChIP H1.2 (blue) and xxChIP H1.5 (green). Also indicated are cytogenetic road marks, chromosome coordinates (mb), the epichromatin track (Olins et al., 2014), and tracks showing the density of peaks enriched with H3K4me1, H3K9ac, H3K27ac, Pol II and SMC3.

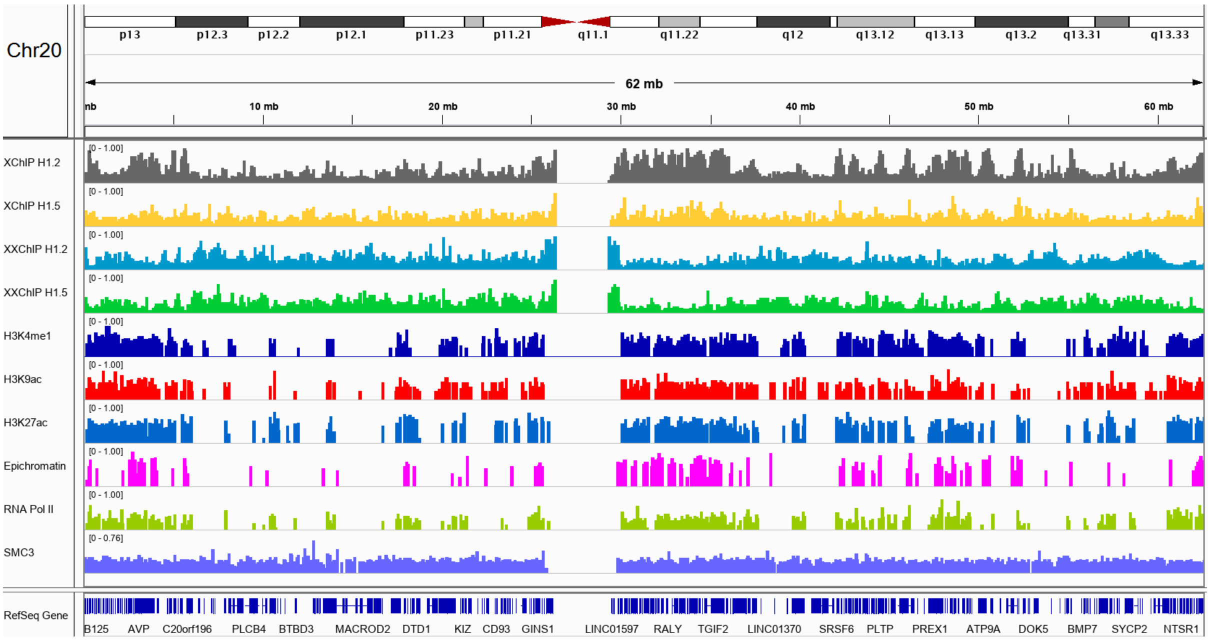

Figure S4. Large-scale (low resolution) comparisons between xChIP and xxChIP sequencing strategies for all the human chromosomes. H1 epitope "exposure" peak densities are measured by scanning with a window of 1000 base pairs: for xChIP H1.2 (black), xChIP H1.5 (orange), xxChIP H1.2 (blue) and xxChIP H1.5 (green). Also indicated are cytogenetic road marks, chromosome coordinates (mb), the epichromatin track (Olins et al., 2014), and tracks showing the density of peaks enriched with H3K4me1, H3K9ac, H3K27ac, Pol II and SMC3.

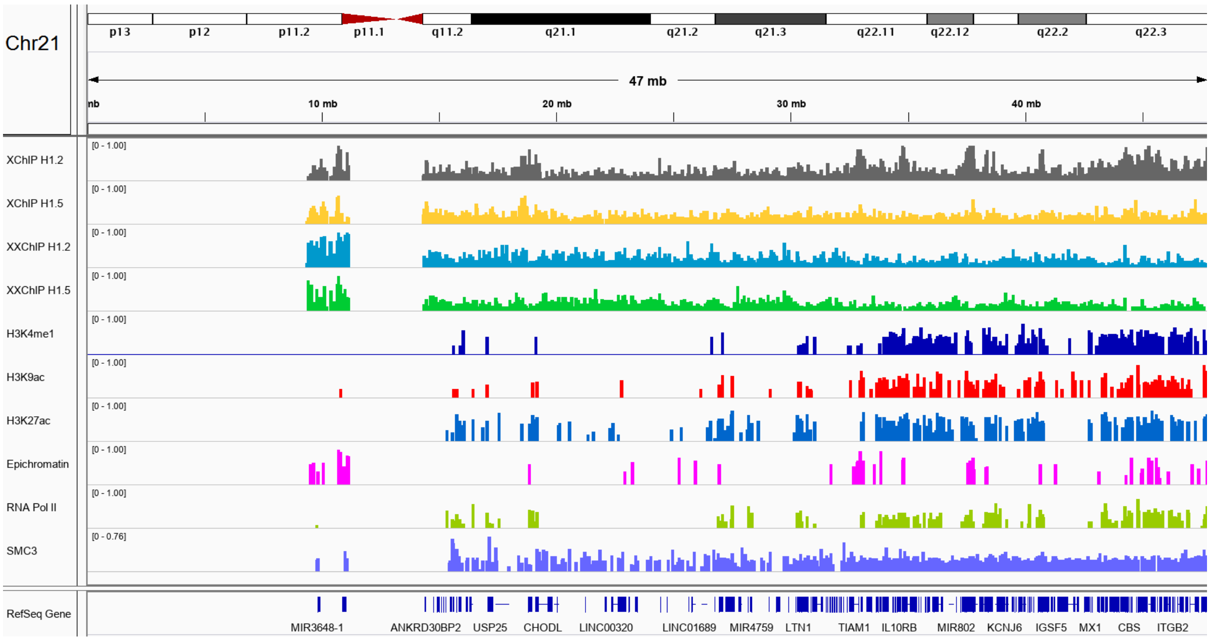

Figure S4. Large-scale (low resolution) comparisons between xChIP and xxChIP sequencing strategies for all the human chromosomes. H1 epitope "exposure" peak densities are measured by scanning with a window of 1000 base pairs: for xChIP H1.2 (black), xChIP H1.5 (orange), xxChIP H1.2 (blue) and xxChIP H1.5 (green). Also indicated are cytogenetic road marks, chromosome coordinates (mb), the epichromatin track (Olins et al., 2014), and tracks showing the density of peaks enriched with H3K4me1, H3K9ac, H3K27ac, Pol II and SMC3.

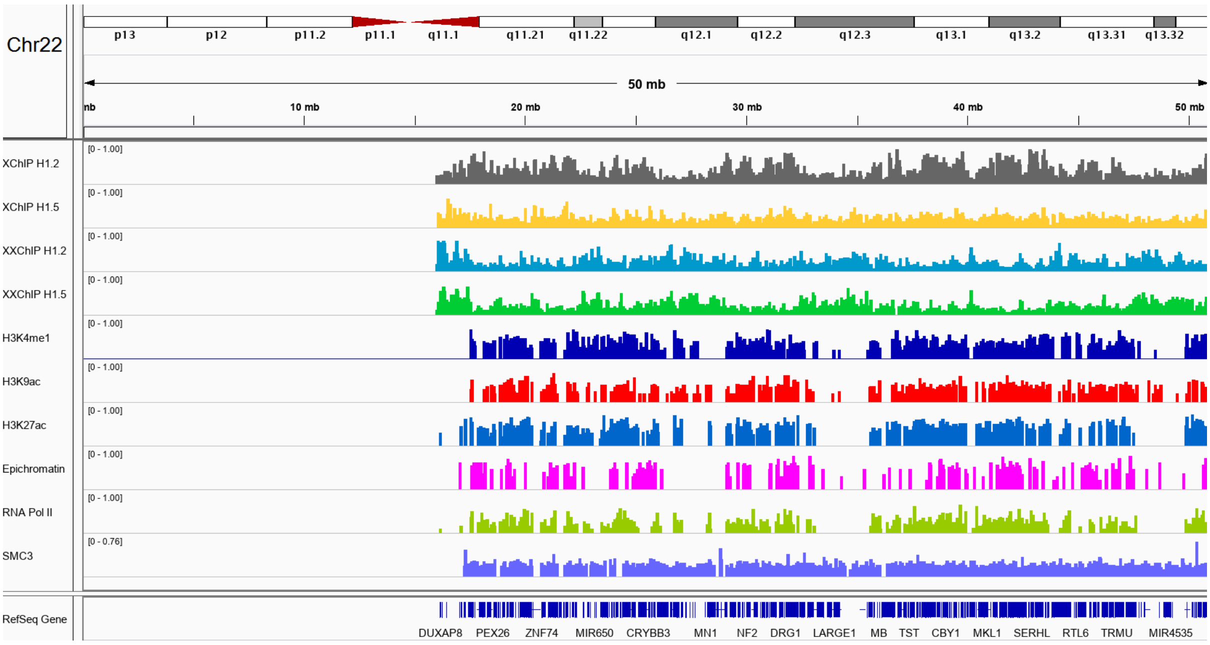

Figure S4. Large-scale (low resolution) comparisons between xChIP and xxChIP sequencing strategies for all the human chromosomes. H1 epitope “exposure” peak densities are measured by scanning with a window of 1000 base pairs: for xChIP H1.2 (black), xChIP H1.5 (orange), xxChIP H1.2 (blue) and xxChIP H1.5 (green). Also indicated are cytogenetic road marks, chromosome coordinates (mb), the epichromatin track (Olins et al., 2014), and tracks showing the density of peaks enriched with H3K4me1, H3K9ac, H3K27ac, Pol II and SMC3.

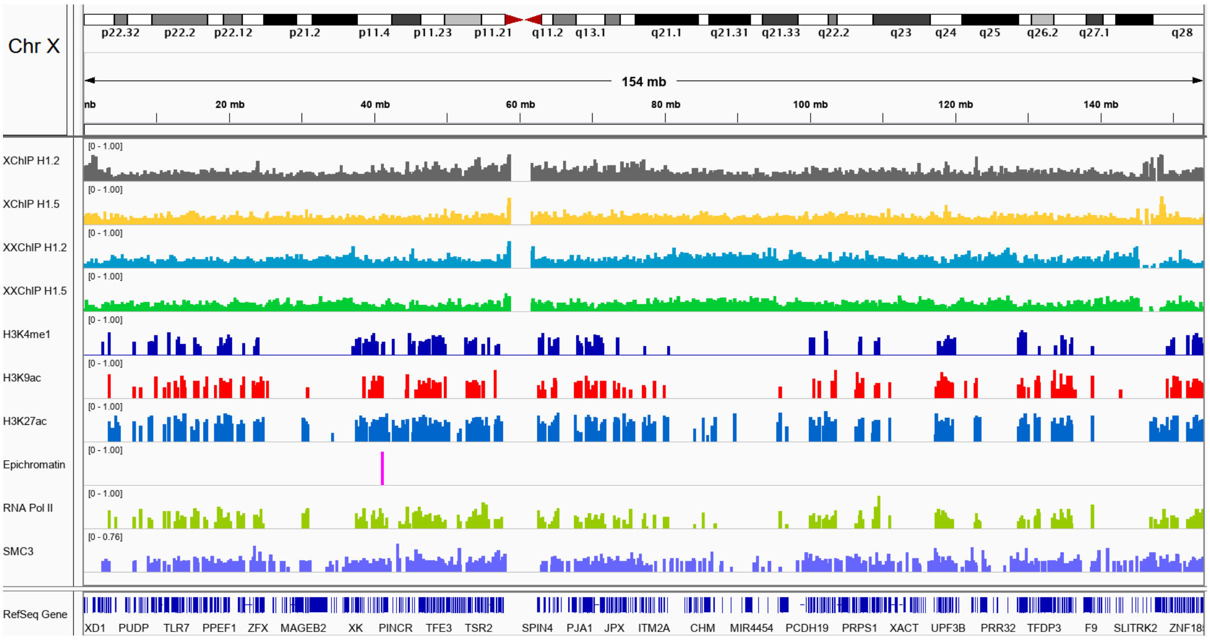

Figure S4. Large-scale (low resolution) comparisons between xChIP and xxChIP sequencing strategies for all the human chromosomes. H1 epitope "exposure" peak densities are measured by scanning with a window of 1000 base pairs: for xChIP H1.2 (black), xChIP H1.5 (orange), xxChIP H1.2 (blue) and xxChIP H1.5 (green). Also indicated are cytogenetic road marks, chromosome coordinates (mb), the epichromatin track (Olins et al., 2014), and tracks showing the density of peaks enriched with H3K4me1, H3K9ac, H3K27ac, Pol II and SMC3.
