# Supplementary material for: Linker histone epitopes are hidden by in situ higher-order chromatin structure
Source: Epigenetics Chromatin. 2020 Jun 6;13:26. doi: 10.1186/s13072-020-00345-9 (PMC7276084; doi:10.1186/s13072-020-00345-9)
Supplement: Supplementary file 2 — Additional file 2: Additional Table S1. [file 13072_2020_345_MOESM2_ESM.pdf]

Table S1. Gene Ontology analysis with DAVID for 100-bp genomic regions where H1.2 xChIP signal dominates over H1.5 xChIP.

Annotation Cluster 1	Enrichment Score: 3.59		Count	P_Value	Benjamini
UP_SEQ_FEATURE	nucleotide phosphate-binding region:ATP	RT	60	8.5E-7	8.6E-4
UP_KEYWORDS	ATP-binding	RT	75	1.3E-6	1.2E-4
GOTERM_MF_DIRECT	protein serine/threonine kinase activity	RT	30	2.6E-5	1.8E-2
UP_KEYWORDS	Serine/threonine-protein kinase	RT	29	2.6E-5	1.2E-3
GOTERM_MF_DIRECT	ATP binding	RT	79	4.6E-5	1.5E-2
UP_KEYWORDS	Nucleotide-binding	RT	83	8.9E-5	3.2E-3
UP_SEQ_FEATURE	domain:Protein kinase	RT	31	1.7E-4	8.4E-2
GOTERM_BP_DIRECT	peptidyl-serine phosphorylation	RT	14	2.8E-4	5.0E-1
UP_KEYWORDS	Transferase	RT	77	3.9E-4	1.1E-2
INTERPRO	Protein kinase-like domain	RT	33	4.6E-4	7.0E-2
INTERPRO	Protein kinase, catalytic domain	RT	31	4.8E-4	6.5E-2
GOTERM_BP_DIRECT	protein phosphorylation	RT	30	7.3E-4	5.9E-1
UP_SEQ_FEATURE	active site:Proton acceptor	RT	37	8.4E-4	1.7E-1

Annotation Cluster 1	Enrichment Score: 3.59	G			Count	P_Value	Benjamini
UP_KEYWORDS	Kinase	RT			39	9.4E-4	2.0E-2
UP_SEQ_FEATURE	binding site:ATP	RT			32	1.1E-3	1.9E-1
INTERPRO	Serine/threonine-protein kinase, active site	RT			22	1.1E-3	1.1E-1
INTERPRO	Protein kinase, ATP binding site	RT			25	1.3E-3	1.1E-1
GOTERM_BP_DIRECT	intracellular signal transduction	RT			26	2.3E-3	6.1E-1
SMART	S_TKc	RT			26	2.8E-3	1.8E-1
GOTERM_MF_DIRECT	protein kinase activity	RT			21	1.8E-2	4.6E-1
**Annotation Cluster 2**	**Enrichment Score: 3.44**	G			**Count**	**P_Value**	**Benjamini**
UP_KEYWORDS	SH3 domain	RT			19	9.8E-5	3.2E-3
INTERPRO	Src homology-3 domain	RT			19	2.3E-4	8.1E-2
UP_SEQ_FEATURE	domain:SH3	RT			16	3.2E-4	1.0E-1
SMART	SH3	RT			18	2.4E-3	4.9E-1
**Annotation Cluster 3**	**Enrichment Score: 2.55**	G			**Count**	**P_Value**	**Benjamini**
GOTERM_MF_DIRECT	microtubule motor activity	RT			12	6.7E-5	1.5E-2
UP_SEQ_FEATURE	region of interest:AAA 6	RT			5	5.0E-4	1.3E-1
UP_SEQ_FEATURE	region of interest:AAA 2	RT			5	6.8E-4	1.6E-1

Annotation Cluster 1		Enrichment Score: 3.59	G			Count	P_Value	Benjamini
	UP_SEQ_FEATURE	region of interest:AAA 3	RT			5	6.8E-4	1.6E-1
	UP_SEQ_FEATURE	region of interest:AAA 1	RT			5	6.8E-4	1.6E-1
	UP_SEQ_FEATURE	region of interest:Stem	RT			5	6.8E-4	1.6E-1
	UP_SEQ_FEATURE	region of interest:Stalk	RT			5	6.8E-4	1.6E-1
	UP_SEQ_FEATURE	region of interest:AAA 5	RT			5	6.8E-4	1.6E-1
	UP_SEQ_FEATURE	region of interest:AAA 4	RT			5	6.8E-4	1.6E-1
	UP_KEYWORDS	Motor protein	RT			13	8.1E-4	1.8E-2
	INTERPRO	Dynein heavy chain, P-loop containing D4 domain	RT			5	1.1E-3	1.1E-1
	GOTERM_MF_DIRECT	ATPase activity	RT			16	1.2E-3	1.9E-1
	INTERPRO	Dynein heavy chain domain	RT			5	1.4E-3	1.1E-1
	INTERPRO	Dynein heavy chain, coiled coil stalk	RT			5	1.4E-3	1.1E-1
	INTERPRO	Dynein heavy chain	RT			5	1.4E-3	1.1E-1
	INTERPRO	Dynein heavy chain, domain-2	RT			5	1.4E-3	1.1E-1
	UP_KEYWORDS	Dynein	RT			6	2.9E-3	4.5E-2
	GOTERM_CC_DIRECT	axonemal dynein complex	RT			4	3.3E-3	2.7E-1
	GOTERM_BP_DIRECT	microtubule-based movement	RT			9	5.6E-3	8.6E-1

Annotation Cluster 1	Enrichment Score: 3.59		Count	P_Value	Benjamini
INTERPRO	ATPase, dynein-related, AAA domain	RT	4	9.0E-3	3.5E-1
UP_KEYWORDS	Microtubule	RT	17	1.1E-2	1.2E-1
GOTERM_CC_DIRECT	microtubule	RT	19	1.2E-2	4.0E-1
INTERPRO	AAA+ ATPase domain	RT	11	1.2E-2	3.9E-1
INTERPRO	Dynein heavy chain, domain-1	RT	3	3.2E-2	6.2E-1
GOTERM_CC_DIRECT	dynein complex	RT	4	3.2E-2	6.1E-1
SMART	AAA	RT	11	3.6E-2	6.8E-1
COG_ONTOLOGY	Cytoskeleton	RT	4	1.1E-1	7.7E-1
KEGG_PATHWAY	Huntington's disease	RT	10	1.7E-1	5.0E-1
**Annotation Cluster 4**	**Enrichment Score: 2.52**		**Count**	**P_Value**	**Benjamini**
INTERPRO	Pleckstrin homology-like domain	RT	30	1.2E-4	1.2E-1
INTERPRO	Pleckstrin homology domain	RT	19	2.8E-3	1.7E-1
SMART	PH	RT	19	1.3E-2	4.6E-1
UP_SEQ_FEATURE	domain:PH	RT	15	1.9E-2	7.2E-1
**Annotation Cluster 5**	**Enrichment Score: 2.37**		**Count**	**P_Value**	**Benjamini**
GOTERM_MF_DIRECT	zinc ion binding	RT	59	1.5E-3	1.6E-1

					Count	P_Value	Benjamini
Annotation Cluster 1		Enrichment Score: 3.59			Count	P_Value	Benjamini
UP_KEYWORDS	Zinc	RT			94	3.6E-3	5.2E-2
UP_KEYWORDS	Zinc-finger	RT			73	6.6E-3	7.7E-2
UP_KEYWORDS	Metal-binding	RT			134	8.9E-3	1.0E-1
Annotation Cluster 6		Enrichment Score: 2.33			Count	P_Value	Benjamini
INTERPRO	WW domain	RT			9	2.6E-4	6.8E-2
SMART	WW	RT			8	2.4E-3	2.9E-1
UP_SEQ_FEATURE	domain:WW 2	RT			5	1.1E-2	6.6E-1
UP_SEQ_FEATURE	domain:WW 1	RT			5	1.1E-2	6.6E-1
UP_SEQ_FEATURE	domain:WW	RT			4	2.8E-2	7.9E-1
Annotation Cluster 7		Enrichment Score: 2.29			Count	P_Value	Benjamini
KEGG_PATHWAY	Ubiquitin mediated proteolysis	RT			13	1.6E-3	1.1E-1
GOTERM_MF_DIRECT	ubiquitin-protein transferase activity	RT			23	1.6E-3	1.5E-1
UP_KEYWORDS	Ubl conjugation pathway	RT			32	1.5E-2	1.5E-1
GOTERM_BP_DIRECT	protein ubiquitination	RT			21	1.8E-2	9.5E-1
Annotation Cluster 8		Enrichment Score: 2.27			Count	P_Value	Benjamini
UP_KEYWORDS	Cell junction	RT			38	3.7E-4	1.1E-2

	Annotation Cluster 1	Enrichment Score: 3.59	G		Count	P_Value	Benjamini
	UP_KEYWORDS	Synapse	RT		23	1.3E-3	2.5E-2
	GOTERM_CC_DIRECT	postsynaptic density	RT		15	2.5E-3	2.5E-1
	GOTERM_CC_DIRECT	cell junction	RT		25	1.3E-2	4.1E-1
	UP_KEYWORDS	Postsynaptic cell membrane	RT		12	2.0E-2	1.8E-1
	GOTERM_CC_DIRECT	postsynaptic membrane	RT		12	7.8E-2	6.8E-1

	Annotation Cluster 9	Enrichment Score: 2.2	G		Count	P_Value	Benjamini
	KEGG_PATHWAY	Morphine addiction	RT		12	1.6E-4	1.8E-2
	INTERPRO	3'5'-cyclic nucleotide phosphodiesterase, conserved site	RT		6	4.3E-4	7.7E-2
	INTERPRO	3'5'-cyclic nucleotide phosphodiesterase, catalytic domain	RT		6	5.4E-4	6.5E-2
	UP_KEYWORDS	cAMP	RT		7	6.6E-4	1.7E-2
	GOTERM_MF_DIRECT	3',5'-cyclic-AMP phosphodiesterase activity	RT		5	1.2E-3	1.6E-1
	GOTERM_BP_DIRECT	cAMP catabolic process	RT		5	1.3E-3	6.4E-1
	UP_SEQ_FEATURE	metal ion-binding site:Divalent metal cation 2	RT		6	1.4E-3	2.3E-1
	UP_SEQ_FEATURE	metal ion-binding site:Divalent metal cation 1	RT		6	1.4E-3	2.3E-1
	INTERPRO	3'5'-cyclic nucleotide phosphodiesterase	RT		5	2.7E-3	1.7E-1

Annotation Cluster 1		Enrichment Score: 3.59			Count	P_Value	Benjamini
	INTERPRO	HD/PDEase domain	RT		5	5.6E-3	2.8E-1
	GOTERM_MF_DIRECT	3',5'-cyclic-nucleotide phosphodiesterase activity	RT		5	6.5E-3	3.6E-1
	SMART	HDc	RT		5	1.1E-2	4.5E-1
	GOTERM_BP_DIRECT	cAMP-mediated signaling	RT		5	3.7E-2	9.8E-1
	UP_SEQ_FEATURE	region of interest:Catalytic	RT		5	7.4E-2	9.2E-1
	KEGG_PATHWAY	Purine metabolism	RT		10	1.2E-1	4.2E-1
	UP_KEYWORDS	cGMP	RT		3	1.3E-1	5.2E-1
	GOTERM_MF_DIRECT	cAMP binding	RT		3	1.9E-1	9.4E-1
	UP_SEQ_FEATURE	active site:Proton donor	RT		8	4.1E-1	1.0E0
