# Supplementary material for: Linker histone epitopes are hidden by in situ higher-order chromatin structure
Source: Epigenetics Chromatin. 2020 Jun 6;13:26. doi: 10.1186/s13072-020-00345-9 (PMC7276084; doi:10.1186/s13072-020-00345-9)
Supplement: Supplementary file 3 — Additional file 3: Additional Table S2. [file 13072_2020_345_MOESM3_ESM.pdf]

Table S2. Gene Ontology analysis with DAVID for 100-bp genomic regions where H1.5 xChIP signal dominates over H1.2 xChIP.

Annotation Cluster 1	Enrichment Score: 7.67	G		Count	P_Value	Benjamini
UP_KEYWORDS	ATP-binding	RT		201	7.7E-14	6.4E-12
UP_KEYWORDS	Nucleotide-binding	RT		242	2.4E-13	1.7E-11
GOTERM_MF_DIRECT	ATP binding	RT		213	1.5E-11	1.0E-8
UP_SEQ_FEATURE	nucleotide phosphate-binding region:ATP	RT		148	3.6E-11	7.8E-8
UP_KEYWORDS	Kinase	RT		114	6.0E-10	3.7E-8
UP_KEYWORDS	Serine/threonine-protein kinase	RT		69	1.7E-8	7.8E-7
GOTERM_BP_DIRECT	protein phosphorylation	RT		80	2.0E-8	4.2E-5
INTERPRO	Protein kinase-like domain	RT		88	2.0E-8	2.3E-5
GOTERM_MF_DIRECT	protein serine/threonine kinase activity	RT		68	6.9E-8	3.1E-5
INTERPRO	Protein kinase, catalytic domain	RT		80	1.4E-7	7.9E-5
UP_SEQ_FEATURE	binding site:ATP	RT		85	3.1E-7	3.3E-4
INTERPRO	Serine/threonine-protein kinase, active site	RT		57	3.5E-7	1.3E-4
UP_SEQ_FEATURE	domain:Protein kinase	RT		73	2.1E-6	1.3E-3
SMART	S_TKc	RT		62	1.2E-5	2.8E-3

						Count	P_Value	Benjamini
**Annotation Cluster 1**		**Enrichment Score: 7.67**	G			**Count**	**P_Value**	**Benjamini**
	**GOTERM_MF_DIRECT**	protein kinase activity	RT			59	1.2E-5	3.4E-3
	**INTERPRO**	Protein kinase, ATP binding site	RT			60	2.2E-5	4.5E-3
	**UP_SEQ_FEATURE**	active site:Proton acceptor	RT			87	1.3E-4	4.2E-2
**Annotation Cluster 2**		**Enrichment Score: 6.98**	G			**Count**	**P_Value**	**Benjamini**
	**INTERPRO**	Pleckstrin homology-like domain	RT			79	8.0E-10	1.8E-6
	**INTERPRO**	Pleckstrin homology domain	RT			53	1.0E-7	7.9E-5
	**UP_SEQ_FEATURE**	domain:PH	RT			46	9.3E-7	6.7E-4
	**SMART**	PH	RT			52	1.5E-6	7.1E-4
**Annotation Cluster 3**		**Enrichment Score: 4.94**	G			**Count**	**P_Value**	**Benjamini**
	**INTERPRO**	AGC-kinase, C-terminal	RT			18	4.6E-6	1.5E-3
	**INTERPRO**	Protein kinase, C-terminal	RT			13	4.7E-6	1.3E-3
	**UP_SEQ_FEATURE**	domain:AGC-kinase C-terminal	RT			17	1.3E-5	7.0E-3
	**SMART**	S_TK_X	RT			17	1.3E-5	2.0E-3
	**GOTERM_BP_DIRECT**	peptidyl-serine phosphorylation	RT			27	5.5E-5	3.8E-2
**Annotation Cluster 4**		**Enrichment Score: 3.91**	G			**Count**	**P_Value**	**Benjamini**
	**UP_KEYWORDS**	Ubl conjugation pathway	RT			99	2.1E-7	8.1E-6

	Annotation Cluster 1	Enrichment Score: 7.67	G			Count	P_Value	Benjamini
☐	GOTERM_MF_DIRECT	ligase activity	RT			47	2.5E-5	4.8E-3
☐	GOTERM_MF_DIRECT	ubiquitin-protein transferase activity	RT			53	6.3E-5	8.4E-3
☐	GOTERM_MF_DIRECT	ubiquitin protein ligase activity	RT			31	1.6E-3	1.2E-1
☐	INTERPRO	Zinc finger, RING-type	RT			36	5.2E-2	7.9E-1
	Annotation Cluster 5	Enrichment Score: 3.65	G			Count	P_Value	Benjamini
☐	INTERPRO	WW domain	RT			16	4.2E-5	7.3E-3
☐	SMART	WW	RT			16	4.3E-5	4.0E-3
☐	UP_SEQ_FEATURE	domain:WW 2	RT			11	9.5E-5	3.4E-2
☐	UP_SEQ_FEATURE	domain:WW 1	RT			11	9.5E-5	3.4E-2
☐	UP_SEQ_FEATURE	domain:WW	RT			6	3.6E-2	8.6E-1
	Annotation Cluster 6	Enrichment Score: 3.31	G			Count	P_Value	Benjamini
☐	UP_KEYWORDS	SH3 domain	RT			37	7.7E-5	1.8E-3
☐	UP_SEQ_FEATURE	domain:SH3	RT			30	4.7E-4	8.2E-2
☐	INTERPRO	Src homology-3 domain	RT			36	4.9E-4	4.9E-2
☐	SMART	SH3	RT			34	3.2E-3	1.0E-1
	Annotation Cluster 7	Enrichment Score: 3.27	G			Count	P_Value	Benjamini

	Annotation Cluster 1	Enrichment Score: 7.67	G			Count	P_Value	Benjamini
	INTERPRO	Zinc finger, A20-type	RT			6	2.5E-4	3.1E-2
	SMART	ZnF_A20	RT			6	3.9E-4	2.2E-2
	UP_SEQ_FEATURE	zinc finger region:A20-type	RT			5	1.6E-3	1.8E-1
	**Annotation Cluster 8**	**Enrichment Score: 3.15**	G			**Count**	**P_Value**	**Benjamini**
	INTERPRO	Protein kinase C-like, phorbol ester/diacylglycerol binding	RT			19	1.3E-5	3.3E-3
	SMART	C1	RT			19	3.5E-5	4.0E-3
	INTERPRO	Diacylglycerol/phorbol-ester binding	RT			10	2.0E-4	2.6E-2
	UP_SEQ_FEATURE	zinc finger region:Phorbol-ester/DAG-type 1	RT			9	3.2E-4	6.4E-2
	UP_SEQ_FEATURE	zinc finger region:Phorbol-ester/DAG-type 2	RT			9	3.2E-4	6.4E-2
	GOTERM_MF_DIRECT	protein kinase C activity	RT			6	8.7E-3	3.5E-1
	UP_SEQ_FEATURE	zinc finger region:Phorbol-ester/DAG-type	RT			10	1.3E-2	6.0E-1
	GOTERM_BP_DIRECT	platelet activation	RT			17	6.0E-2	9.8E-1
	**Annotation Cluster 9**	**Enrichment Score: 2.93**	G			**Count**	**P_Value**	**Benjamini**
	UP_KEYWORDS	Guanine-nucleotide releasing factor	RT			31	8.3E-6	2.6E-4
	UP_SEQ_FEATURE	domain:Ras-GEF	RT			11	9.5E-5	3.4E-2
	INTERPRO	Ras guanine nucleotide exchange factor, domain	RT			11	1.8E-4	2.5E-2

Annotation Cluster 1	Enrichment Score: 7.67		Count	P_Value	Benjamini
INTERPRO	Guanine-nucleotide dissociation stimulator CDC25	RT	11	1.8E-4	2.5E-2
SMART	RasGEF	RT	11	3.8E-4	2.9E-2
UP_SEQ_FEATURE	domain:N-terminal Ras-GEF	RT	8	1.9E-3	2.0E-1
SMART	RasGEFN	RT	8	2.1E-3	7.8E-2
GOTERM_MF_DIRECT	guanyl-nucleotide exchange factor activity	RT	22	2.2E-3	1.5E-1
INTERPRO	Ras-like guanine nucleotide exchange factor, N-terminal	RT	8	2.3E-3	1.4E-1
INTERPRO	Ras guanine-nucleotide exchange factor, conserved site	RT	6	5.7E-3	2.7E-1
GOTERM_MF_DIRECT	Rap guanyl-nucleotide exchange factor activity	RT	3	6.9E-2	7.7E-1
GOTERM_BP_DIRECT	small GTPase mediated signal transduction	RT	30	9.5E-2	9.9E-1
**Annotation Cluster 10**	**Enrichment Score: 2.88**		**Count**	**P_Value**	**Benjamini**
GOTERM_MF_DIRECT	GTPase activator activity	RT	47	5.7E-5	9.6E-3
GOTERM_BP_DIRECT	regulation of small GTPase mediated signal transduction	RT	26	4.5E-4	1.6E-1
INTERPRO	Rho GTPase-activating protein domain	RT	15	2.1E-3	1.3E-1
INTERPRO	Rho GTPase activation protein	RT	18	2.7E-3	1.6E-1
UP_KEYWORDS	GTPase activation	RT	30	2.9E-3	3.4E-2

				Count	P_Value	Benjamini
**Annotation Cluster 1**	**Enrichment Score: 7.67**	G		**Count**	**P_Value**	**Benjamini**
SMART	RhoGAP	RT		15	3.6E-3	1.0E-1
UP_SEQ_FEATURE	domain:Rho-GAP	RT		14	4.9E-3	3.7E-1
**Annotation Cluster 11**	**Enrichment Score: 2.8**	G		**Count**	**P_Value**	**Benjamini**
UP_KEYWORDS	Metal-binding	RT		380	1.1E-5	3.2E-4
UP_KEYWORDS	Zinc-finger	RT		187	2.3E-3	3.0E-2
GOTERM_MF_DIRECT	zinc ion binding	RT		130	1.2E-2	3.8E-1
UP_KEYWORDS	Zinc	RT		229	2.2E-2	1.5E-1
**Annotation Cluster 12**	**Enrichment Score: 2.66**	G		**Count**	**P_Value**	**Benjamini**
GOTERM_MF_DIRECT	3',5'-cyclic-AMP phosphodiesterase activity	RT		9	1.7E-5	3.8E-3
GOTERM_BP_DIRECT	cAMP catabolic process	RT		9	1.7E-5	1.8E-2
INTERPRO	3'5'-cyclic nucleotide phosphodiesterase, conserved site	RT		10	3.9E-5	7.3E-3
INTERPRO	3'5'-cyclic nucleotide phosphodiesterase, catalytic domain	RT		10	6.1E-5	9.8E-3
INTERPRO	3'5'-cyclic nucleotide phosphodiesterase	RT		9	1.2E-4	1.8E-2
UP_SEQ_FEATURE	metal ion-binding site:Divalent metal cation 1	RT		10	3.9E-4	7.4E-2
UP_SEQ_FEATURE	metal ion-binding site:Divalent metal cation 2	RT		10	3.9E-4	7.4E-2
KEGG_PATHWAY	Morphine addiction	RT		20	4.7E-4	1.2E-2

Annotation Cluster 1	Enrichment Score: 7.67	G			Count	P_Value	Benjamini
INTERPRO	HD/PDEase domain	RT			9	5.8E-4	5.3E-2
UP_KEYWORDS	cAMP	RT			11	6.7E-4	1.1E-2
SMART	HDc	RT			9	1.1E-3	4.8E-2
UP_SEQ_FEATURE	region of interest:Catalytic	RT			13	1.3E-3	1.6E-1
GOTERM_MF_DIRECT	3',5'-cyclic-nucleotide phosphodiesterase activity	RT			8	3.4E-3	2.2E-1
GOTERM_MF_DIRECT	cAMP binding	RT			7	1.8E-2	4.5E-1
UP_SEQ_FEATURE	active site:Proton donor	RT			27	2.3E-2	7.4E-1
UP_KEYWORDS	cGMP	RT			6	2.9E-2	1.8E-1
GOTERM_MF_DIRECT	3',5'-cyclic-GMP phosphodiesterase activity	RT			5	3.3E-2	5.7E-1
UP_SEQ_FEATURE	site:Binds AMP, but not cAMP	RT			3	4.0E-2	8.7E-1
KEGG_PATHWAY	Purine metabolism	RT			24	5.0E-2	2.0E-1
UP_SEQ_FEATURE	binding site:cAMP	RT			3	8.8E-2	9.7E-1
GOTERM_BP_DIRECT	cGMP catabolic process	RT			3	1.3E-1	9.9E-1
UP_SEQ_FEATURE	nucleotide phosphate-binding region:cAMP	RT			3	2.4E-1	1.0E0
**Annotation Cluster 13**	**Enrichment Score: 2.63**	**G**			**Count**	**P_Value**	**Benjamini**
GOTERM_MF_DIRECT	microtubule binding	RT			36	2.8E-4	3.1E-2

Annotation Cluster 1	Enrichment Score: 7.67	G		Count	P_Value	Benjamini
GOTERM_CC_DIRECT	microtubule	RT		43	4.8E-3	2.0E-1
UP_KEYWORDS	Microtubule	RT		37	9.4E-3	7.9E-2
**Annotation Cluster 14**	**Enrichment Score: 2.49**	**G**		**Count**	**P_Value**	**Benjamini**
UP_SEQ_FEATURE	repeat:ANK 10	RT		10	5.2E-4	8.7E-2
UP_SEQ_FEATURE	repeat:ANK 23	RT		6	8.2E-4	1.2E-1
UP_SEQ_FEATURE	repeat:ANK 22	RT		6	8.2E-4	1.2E-1
UP_SEQ_FEATURE	repeat:ANK 11	RT		9	8.5E-4	1.2E-1
UP_SEQ_FEATURE	repeat:ANK 13	RT		7	1.0E-3	1.3E-1
UP_SEQ_FEATURE	repeat:ANK 14	RT		7	1.0E-3	1.3E-1
UP_SEQ_FEATURE	repeat:ANK 15	RT		7	1.0E-3	1.3E-1
UP_SEQ_FEATURE	repeat:ANK 20	RT		6	1.4E-3	1.7E-1
UP_SEQ_FEATURE	repeat:ANK 21	RT		6	1.4E-3	1.7E-1
UP_SEQ_FEATURE	repeat:ANK 8	RT		12	1.5E-3	1.7E-1
UP_SEQ_FEATURE	repeat:ANK 17	RT		6	2.2E-3	2.3E-1
UP_SEQ_FEATURE	repeat:ANK 18	RT		6	2.2E-3	2.3E-1
UP_SEQ_FEATURE	repeat:ANK 19	RT		6	2.2E-3	2.3E-1

Annotation Cluster 1	Enrichment Score: 7.67		Count	P_Value	Benjamini
UP_SEQ_FEATURE	repeat:ANK 7	RT	14	2.4E-3	2.4E-1
UP_SEQ_FEATURE	repeat:ANK 24	RT	5	2.9E-3	2.7E-1
UP_SEQ_FEATURE	repeat:ANK 12	RT	7	3.0E-3	2.8E-1
UP_SEQ_FEATURE	repeat:ANK 16	RT	6	3.4E-3	2.9E-1
UP_KEYWORDS	ANK repeat	RT	37	3.7E-3	4.2E-2
UP_SEQ_FEATURE	repeat:ANK 4	RT	26	5.8E-3	4.0E-1
UP_SEQ_FEATURE	repeat:ANK 9	RT	10	7.1E-3	4.5E-1
INTERPRO	Ankyrin repeat	RT	36	7.2E-3	3.0E-1
INTERPRO	Ankyrin repeat-containing domain	RT	37	7.7E-3	3.1E-1
UP_SEQ_FEATURE	repeat:ANK 5	RT	22	1.0E-2	5.4E-1
UP_SEQ_FEATURE	repeat:ANK 6	RT	17	1.0E-2	5.4E-1
UP_SEQ_FEATURE	repeat:ANK 1	RT	33	1.1E-2	5.5E-1
UP_SEQ_FEATURE	repeat:ANK 3	RT	29	1.1E-2	5.5E-1
UP_SEQ_FEATURE	repeat:ANK 2	RT	33	1.2E-2	5.6E-1
UP_SEQ_FEATURE	repeat:ANK 25	RT	4	1.7E-2	6.9E-1
SMART	ANK	RT	35	2.9E-2	4.1E-1
